# A Disulfide‐Sticker Strategy for Marine Adhesive Coatings: From Deciphering Self‐Assembly Mechanism to Functional Application in Hair Regeneration

**DOI:** 10.1002/advs.76497

**Published:** 2026-07-09

**Authors:** Lulu Wang, Juan Yang, Xin Jiang, Zhanghui Zheng, Hongyu Wei, Na Li, Xiangqiang Chu, Weizhi Liu

**Affiliations:** ^1^ Fang Zongxi Center for Marine Evo‐Devo, MoE Key Laboratory of Marine Genetics and Breeding, College of Marine Life Sciences Ocean University of China Qingdao China; ^2^ Laboratory for Marine Biology and Biotechnology Qingdao National Laboratory For Marine Science and Technology Qingdao China; ^3^ Department of Physics City University of Hong Kong Hong Kong China; ^4^ Shenzhen Research Institute City University of Hong Kong Shenzhen China; ^5^ Neutron Science Platform Songshan Lake Materials Laboratory Dongguan China; ^6^ National Facility for Protein Science Shanghai Chinese Academy of Sciences (CAS) Shanghai China

**Keywords:** disulfide bonds, hair regeneration, self‐assembly, thiols, underwater adhesion

## Abstract

Marine adhesive organisms commonly employ epidermal growth factor (EGF)‐like domains for wet attachment, yet the molecular mechanisms guiding their self‐assembly remain elusive. Here, we report a disulfide‑sticker strategy in the recombinant scallop adhesive protein Sbp9^Δ^. Dynamic disulfide bonds, acting synergistically with Ca^2+^ coordination, orchestrate the multiscale hierarchical self‐assembly of Sbp9^Δ^ by modulating its conformational heterogeneity. Spectroscopic and scattering analyses reveal that disulfide formation acts as a covalent sticker, rigidifying Sbp9^Δ^ into β‐sheet‐rich rod‐like nanostructures, which direct orderly aggregation into extensive two‐dimensional networks. The resulting coating exhibits robust wet adhesion across diverse substrates, accompanied by intrinsic antioxidant activity. As a proof of concept, the biocompatible Sbp9^Δ^ coating markedly promotes hair regeneration by enhancing angiogenesis, stimulating follicular cell proliferation, and effectively scavenging reactive oxygen species (ROS), exhibiting superior efficacy compared with minoxidil. In a mouse model of androgenetic alopecia, the Sbp9^Δ^ coating activates the follicular niche through the upregulation of Wnt signaling and the downregulation of calcium signaling, leading to robust hair follicle activation. By integrating insights from marine biology, biophysics, and materials science, this work elucidates a disulfide‐mediated assembly paradigm in marine adhesives and translates it into a functional strategy for hair regeneration.

## Graphical abstract

A disulfide‐sticker strategy, coupled with Ca^2+^ coordination, drives the hierarchical self‐assembly of a recombinant scallop adhesive protein into fishnet‐like nanostructures, affording a coating with versatile wet adhesion and intrinsic antioxidant activity. This biocompatible coating markedly promotes hair regeneration by activating follicular niches, enhancing angiogenesis, and scavenging ROS, outperforming minoxidil in an androgenetic alopecia model.

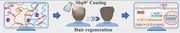

## 1 Introduction

Androgenetic alopecia (AGA), the most prevalent form of non‑scarring hair loss, affects up to 74.8% of men and a significant proportion of women, imposing a considerable physical and psychological burden [1]. Its pathogenesis is mainly driven by dihydrotestosterone accumulation within hair follicles, which inhibits the proliferation of dermal papilla stem cells and leads to progressive follicular miniaturization [2, 3]. An adverse perifollicular microenvironment, marked by oxidative stress and inadequate angiogenesis, further impairs the telogen‐to‐anagen transition [4]. As a cytotoxic event, oxidative stress generates excessive reactive oxygen species (ROS), causing damage to lipids, proteins, and DNA, and ultimately driving hair follicles toward permanent growth arrest [5]. Current clinical pharmacotherapies, such as minoxidil and finasteride, offer partial efficacy and are constrained by interindividual variability and side effects, including cutaneous irritation [6]. Consequently, research has increasingly focused on nanomaterial strategies, employing engineered microneedles, liposomes, and nanoparticles to deliver antioxidants like curcumin [7], resveratrol [8], and quercetin [9], aiming to reduce perifollicular oxidative stress. For instance, a microneedle patch encapsulating iron‐chelating puerarin/quercetin nanoparticles was developed and effectively scavenged follicular ROS and upregulated hair growth‐promoting genes, achieving improved hair coverage [10]. Nonetheless, such nanomaterials face challenges in long‐term stability and low bioavailability [11]. Therefore, developing proactive antioxidant strategies capable of directly remodeling the detrimental perifollicular microenvironment represents a pressing and unmet therapeutic need for AGA.

**SCHEME 1:**
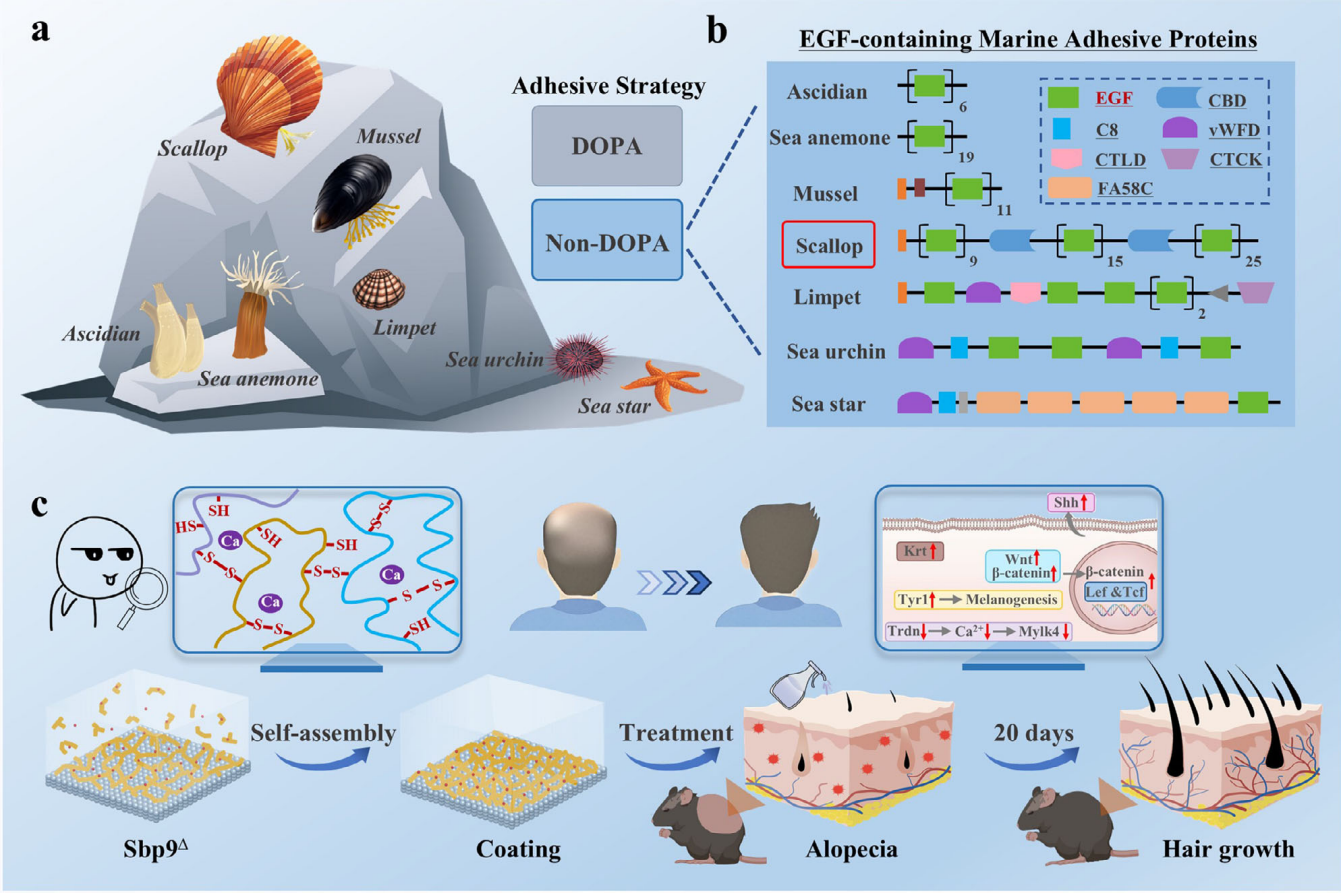
Marine adhesion‐inspired disulfide‐sticker strategies for functional coatings. (a) Marine sessile organisms adhere in wet environments. (b) Many species, such as ascidians, sea anemones, mussels, scallops, limpets, sea urchins, and sea stars, secrete adhesives containing EGF‐like domains, representing an unconventional, non‐Dopa assembly pattern. (c) The scallop adhesive protein Sbp9^∆^, composed of four EGF‐like repeats, can achieve hierarchical self‐assembly through a dual coordination mode involving Ca^2+^ and disulfide bonds, forming interfacial fishnet‐like coatings. When translated into biomedical applications, the Sbp9^∆^ coating successfully stimulates hair regeneration in AGA mice by triggering the telogen‐to‐anagen transition.

Inspired by nature, marine sessile organisms such as mussels [12], barnacles [13], sandcastle worms [14], and sea anemones [15] exhibit robust underwater adhesion on diverse substrates through protein secretion. This has motivated the design of programmable protein‐based biomaterials. Given their good biocompatibility and tunable self‐assembly properties, such biomaterials have demonstrated wide applicability in hemostasis, wound repair, and bone regeneration [16]. Among natural adhesives, the mussel‐derived molecule 3,4‐dihydroxyphenylalanine (Dopa) has been the most intensively studied. Nonetheless, its practical utility is hindered by susceptibility to oxidation and complex synthesis processes, spurring interest in alternative, non‐Dopa adhesion mechanisms [17, 18]. Notably, epidermal growth factor (EGF)‐like domains are ubiquitous components of many marine adhesives (Scheme 1), including mussel Mfp2 [19], sea star Sfp1 [20], and scallop Sbp9 [21]. The spatial arrangement and repetition number of EGF‐like domains critically influence the microstructure and interfacial adsorption of the resulting adhesives or coatings. For example, in sea star Sfp1, Ca^2+^/Mg^2+^ mediates the formation of dense coatings via EGF‐like subunits [22], whereas in mussel Mefp‐2, interactions between EGF‐like domains and N‑acetyl‑D‑glucosamine (GlcNAc) enable a threefold increase in adhesion force compared to Mfp5 [23]. Structurally, EGF‐like domains typically contain six conserved cysteine (Cys) residues, which can form either disulfide bonds or exist as free thiols [24]. Disulfide bonds are known to drive intra‐ and intermolecular crosslinking, thereby enhancing structural integrity [25]. However, the precise molecular pathways and mechanisms by which disulfide bonds dynamically regulate the conformational evolution, microstructure, and interfacial adhesion of marine EGF‑like domains remain to be elucidated. Deciphering this regulatory mechanism will not only deepen our understanding of marine bioadhesion but also provide a blueprint for the rational design of next‐generation biomimetic materials.

Scallops, similar to mussels, adhere to substrates through byssus secretion [26]. Our previous work identified a family of scallop byssus proteins (Sbps) and characterized a chimeric protein, Sbp9, which comprises calcium‐binding domains and EGF‐like repeats [27, 28]. A recombinant CE4 protein derived from Sbp9 self‐assembles into biocompatible hydrogels capable of encapsulating doxorubicin, effectively killing tumor cells and demonstrating considerable potential for drug delivery [29]. Furthermore, a recombinant Sbp9^Δ^ protein rapidly forms extracellular matrix (ECM)‐mimetic coatings through Ca^2+^‐mediated self‐assembly, exhibiting strong wet stability along with antibacterial, anti‐inflammatory, and antioxidant activities. In animal models, Sbp9^Δ^ coatings promoted wound healing by scavenging ROS and driving M2 macrophage polarization, thereby accelerating re‐epithelialization [21]. Given its propensity for in situ assembly and intrinsic bioactivity, we hypothesized that this marine adhesive protein coating could represent a novel therapeutic strategy for AGA. Moving beyond the established role of coordination bonds, we herein report that dynamic disulfide bonds function as essential molecular locks during Sbp9^Δ^ self‐assembly and coating formation, critically facilitating protein crosslinking and reinforcing interfacial adhesion (Scheme 1). As a proof of concept, application of the Sbp9^Δ^ coating significantly enhanced hair regeneration in a mouse model of AGA by ameliorating the hostile perifollicular microenvironment, underscoring its translational potential for regenerative medicine.

## 2 Results and Discussion

### 2.1 Roles of EGF‐Like Repeats and Ca^2+^ in Coating Formation

**FIGURE 1:**
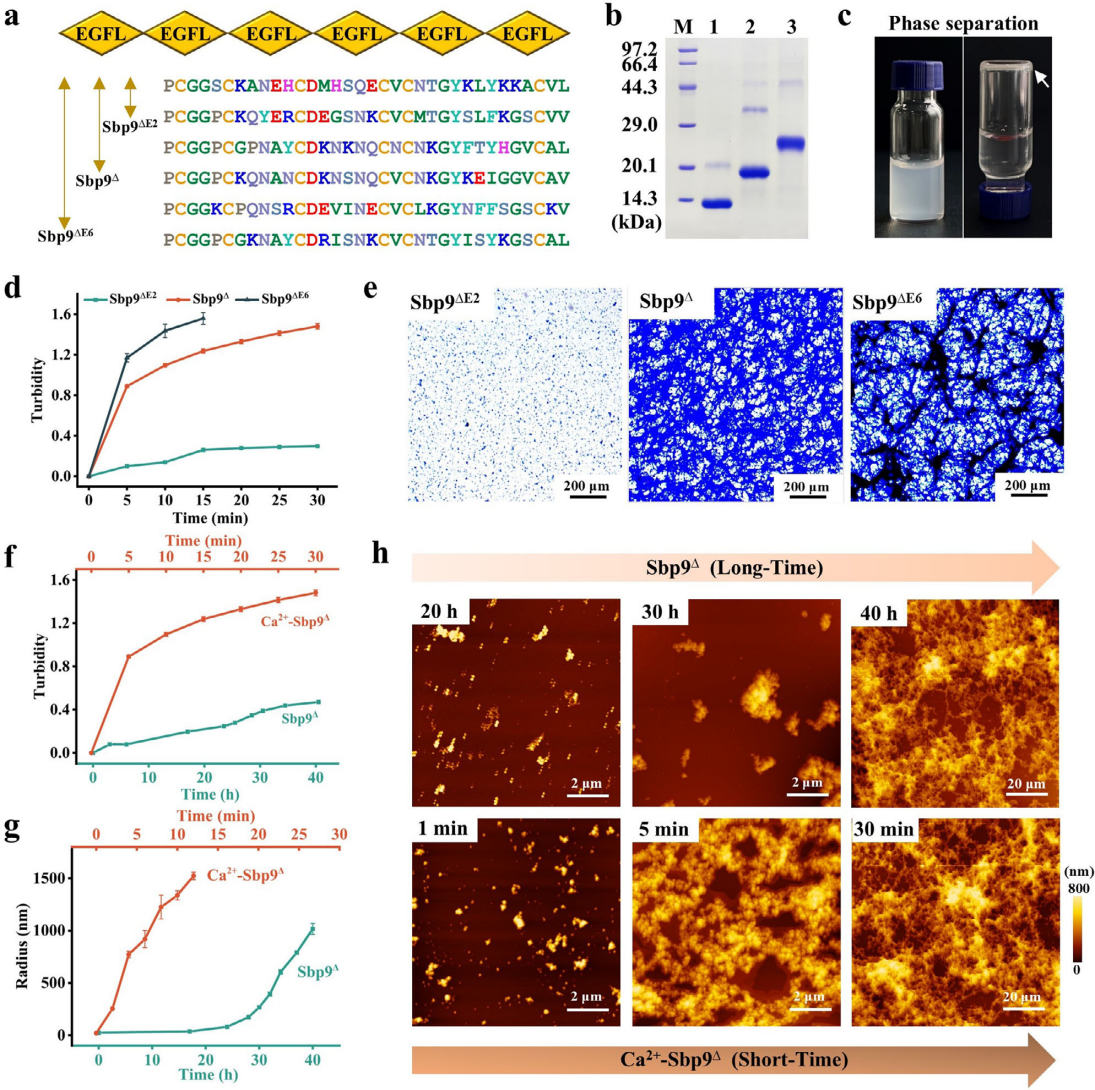
EGF‐like repeats and Ca^2+^ regulate the self‐assembly of recombinant scallop adhesive proteins. (a) Domain composition and amino acid sequences of the Sbp9^∆E2^, Sbp9^∆^, and Sbp9^∆E6^ proteins. (b) Purity analysis of the recombinant proteins via SDS‐PAGE. M, protein marker. Lane 1, Sbp9^∆E2^. Lane 2, Sbp9^∆^. Lane 3, Sbp9^∆E6^. (c) Ca^2+^‐induced phase separation of Sbp9^∆^, with the dense phase indicated by a white arrow. (d) Turbidity changes during the Ca^2+^‐triggered self‐assembly of proteins containing different numbers of EGF‐like repeats. (e) Microstructures of coatings formed by Sbp9^∆E2^, Sbp9^∆^, and Sbp9^∆E6^. (f) Turbidity and (g) hydrodynamic radius (R_h_) of Sbp9^∆^ assemblies in the presence and absence of Ca^2+^. (h) Morphology of aggregates formed under Ca^2+^‑containing and Ca^2+^‑free conditions.

The full‐length Sbp9 contains 49 EGF‐like repeats with a molecular weight exceeding 200 kDa, making studies of the native‐size protein technically challenging. To address this, truncated constructs with fewer EGF‐like repeats provide a feasible and informative approach for dissecting the molecular mechanism of self‐assembly. We previously engineered a recombinant scallop adhesive protein, containing four EGF‐like repeats, which undergoes Ca^2+^‐triggered self‐assembly into dense, network‐like coatings with strong wet adsorption [21]. Here, additional constructs with two and six EGF‐like repeats were designed, designated Sbp9^∆E2^ and Sbp9^∆E6^, respectively (Figure 1a). All proteins were expressed in prokaryotic systems and purified from inclusion bodies, yielding products with purities exceeding 85% (Figure 1b).

Upon Ca^2+^ induction, the Sbp9^∆^ rapidly increased in optical turbidity and underwent phase separation, with the dense phase firmly adhering to glass to form coherent coatings (Figure 1c). We systematically compared the solution self‐assembly behavior and resulting coating microstructure among the three constructs. As shown in Figure 1d, turbidity measurements revealed a clear dependence on EGF‐like repeat number. Sbp9^∆E2^ exhibited minimal self‐assembly, with a turbidity of 0.3 at 30 min, whereas Sbp9^∆E6^ reached 1.6 at 15 min. Dynamic light scattering (DLS) further supported this trend, with the hydrodynamic radius (R_h_) of Sbp9^∆E6^ aggregates reaching 1,353 nm within 4 min, compared to only 204 nm for Sbp9^∆E2^ at 30 min (Figure S1). Sbp9^∆^ exhibited intermediate turbidity and R_h_ values. Microscopic examination of the formed coatings showed that Sbp9^∆E2^ produced sparse, incomplete layers, while Sbp9^∆E6^ displayed excessive tangling. Only Sbp9^∆^ formed a uniform, ordered fishnet‐like microstructure (Figure 1e). These results indicate that more EGF‐like repeats accelerate self‐assembly, likely due to increased exposure of Ca^2+^‐binding sites that promote inter‐framework crosslinking. Similarly, in the aciniform spidroin AcSp1, an increase in repeat units accelerates phase separation and leads to larger droplets [30]. However, excessive repeats can lead to overly rapid aggregation in the scallop adhesive protein, resulting in large, disordered clusters that compromise coating homogeneity. Thus, an optimal repeat number, exemplified by the four‐repeat‐containing Sbp9^∆^, balances assembly dynamics with structural regularity, yielding a mechanically robust, adhesive network.

To clarify the role of Ca^2+^ in assembly, we characterized Sbp9^∆^ behavior in a Ca^2+^‐free environment. Phase separation completed within 30 min in the presence of Ca^2+^, while nucleation and growth only occurred after 40 h without Ca^2+^ (Figure S2). Turbidity and R_h_ measurements provided further evidence that the Ca^2+^‐free group required substantially longer to reach the nucleation plateau, with a turbidity of 0.47 and R_h_ of 1,017 nm at 40 h (Figure 1f,g). Atomic force microscope (AFM) imaging showed that with Ca^2+^, densely packed nanospheres formed within 5 min, whereas without Ca^2+^, aggregates appeared only after 30 h, eventually yielding a porous, network‐like structure (Figure 1h). These data demonstrate that Ca^2+^‐mediated coordination bonds markedly accelerate Sbp9^∆^ self‐assembly, reducing the required time from 40 h to 30 min.

Taken together, these findings elucidate the synergistic role of EGF‐like repeats and Ca^2+^ coordination in governing the morphological order and structural integrity of coatings. Similar to aciniform spidroin AcSp1, an increase in repetitive units amplified aggregation propensity and led to the formation of larger droplets due to enhanced multivalent interactions [30]. Notably, metal ion coordination further augmented recombinant subunit self‐assembly, acting as a molecular zipper that directs crosslinking and facilitates higher‐order architecture formation [22]. The synergy between modular repeats and ion triggers may represent a generalizable design strategy for engineering nanomaterials with programmable assembly pathways and customizable structural features.

### 2.2 Redox‐Tunable Sbp9^∆^ Interfacial Adhesion

**FIGURE 2:**
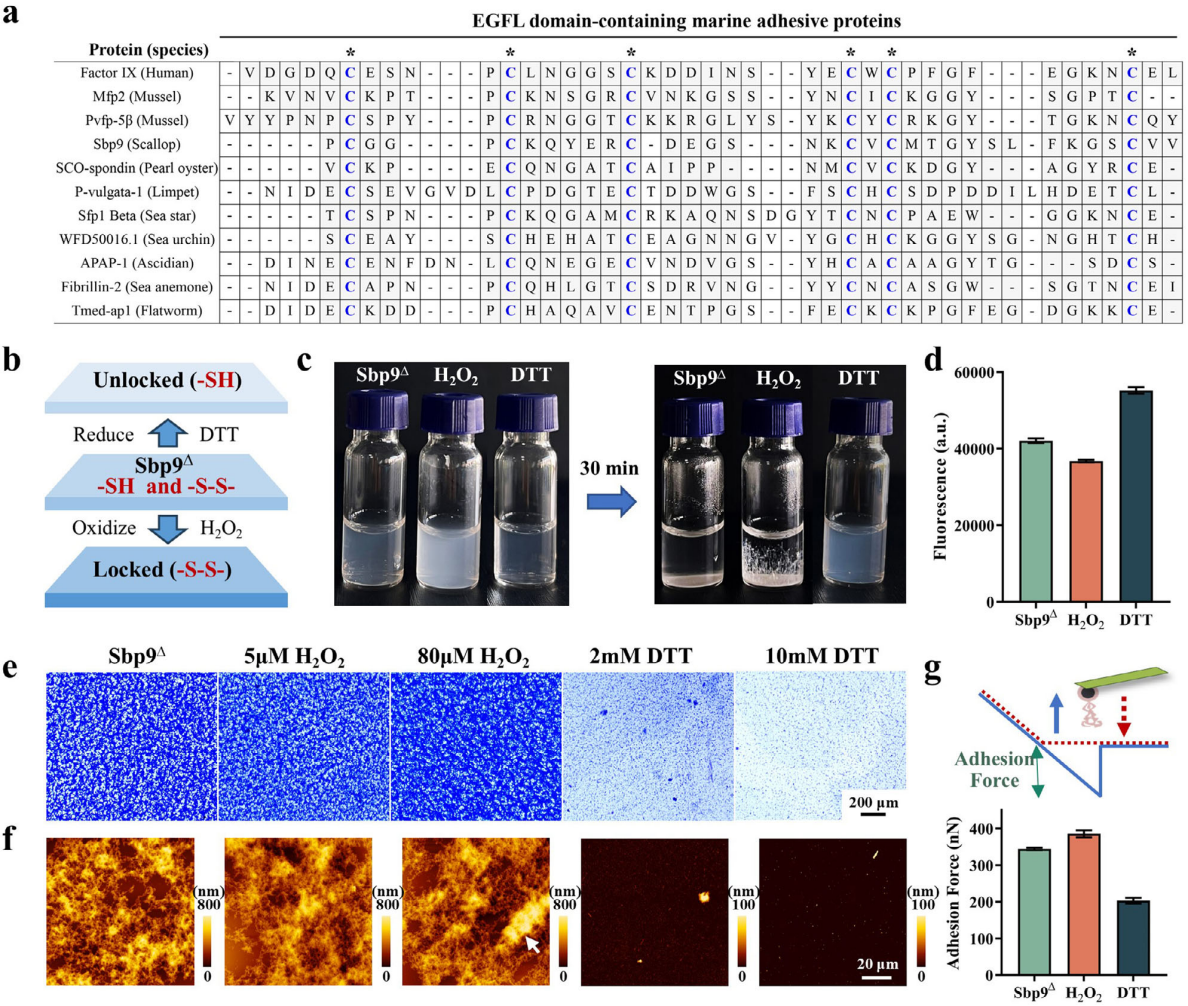
Dynamic disulfide bonds orchestrate the formation, morphology, and interfacial adhesion of Sbp9^∆^ coatings. (a) Multiple sequence alignment of EGF‐like domains from human and marine adhesive organisms. Asterisks mark the six conserved Cys residues. (b) Schematic of the Sbp9^∆^ redox state, containing both disulfide bonds and free thiols, and its modulation by H_2_O_2_ and DTT, which respectively promote disulfide formation and thiol reduction. (c) Redox environment influences Sbp9^∆^ phase separation. H_2_O_2_ treatment yields abundant coatings on glass, whereas DTT prevents coating formation. (d) Fluorescence intensity of NPM‐labeled Sbp9^∆^ solutions. (e) Visual staining and (f) AFM images of Sbp9^∆^ coatings formed under different H_2_O_2_ or DTT concentrations. (g) Adhesion force of Sbp9^∆^ coatings on silicon measured by AFM.

The ability of Sbp9^∆^ to self‐assemble even in the absence of Ca^2+^ implies that additional drivers, beyond coordination bonds, contribute to aggregation. To identify such intrinsic regulators, we conducted a multiple sequence alignment of EGF‐like domains across species, including human coagulation factor IX and marine adhesive organisms such as mussels [19, 24], scallops [21], pearl oysters [31], limpets [32], sea stars [20], sea urchins [33], ascidians [34], sea anemones [15], and flatworms [35]. Although six Cys residues are highly conserved across EGF‐like domains, their functional roles diverge considerably (Figure 2a). In human fibrillin and coagulation factor IX, all six Cys form three structural disulfide bonds essential for domain stability [36, 37]. In contrast, studies on scallop Sbp9^∆^ reveal that not all Cys participate in disulfide bonding, with a proportion remaining as solvent‐accessible thiols [28]. Furthermore, the functional versatility of EGF‐like domains in marine adhesives is well illustrated by other model proteins. For instance, mussel Mefp‐2 utilizes EGF‐like repeats to interact with polysaccharides, whereas sea star Sfp1 employs them to coordinate divalent metal ions, thereby enhancing coating density at interfaces [23, 38]. Nevertheless, the mechanistic basis of redox‐mediated self‐assembly, specifically, how dynamic disulfide bonds or free thiols contribute to interfacial adhesion and cohesion, remains incompletely understood.

Cys residues constitute 17.6% of the total amino acids in Sbp9^∆^, rendering the protein intrinsically sensitive to redox conditions (Figure S3). Free thiols can be oxidized to disulfide bonds under oxidizing conditions, whereas pre‐existing disulfide bonds can be disrupted to free thiols by reducing agents (Figure 2b). To delineate the unique role of disulfide bonding in Sbp9^∆^ coating formation, we treated Sbp9^∆^ solutions with either the oxidant hydrogen peroxide (H_2_O_2_) or the reductant dithiothreitol (DTT). As shown in Figure 2c, H_2_O_2_ treatment increased solution turbidity and enhanced the deposition of a continuous coating on glass surfaces. Conversely, DTT suppressed phase separation, indicating that Sbp9^∆^ self‐assembly is regulated by redox conditions. Using the thiol‐sensitive N‐(1‐pyrenyl) maleimide (NPM) dye, a sharp increase in fluorescence intensity was observed following DTT treatment, confirming rapid disulfide reduction and the consequent exposure of free thiols (Figure 2d) [39]. We further characterized the morphological and interfacial adhesive properties of the resulting coatings. Staining and AFM images revealed that treatment with 80 µM H_2_O_2_ yielded a thickened coating, whereas DTT disrupted this organized architecture, resulting in scattered spherical aggregates (Figure 2e,f). Consistent with these structural changes, the adhesion force of the coating increased from 345 nN to 386 nN with 80 µM H_2_O_2_ but dropped to 203 nN with 2 mM DTT (Figure 2g and Figure S4). Taken together, oxidative conditions enhance coating compactness and interfacial adhesion through disulfide crosslinking, whereas reductive cleavage of these bonds severely compromises both the structural integrity and functional performance of the material. This redox‐switchable behavior underscores the critical role of dynamic covalent chemistry in tuning the assembly and mechanics of Sbp9^∆^‐based bioadhesives.

### 2.3 Ordered Self‐Assembly Governed by Disulfide‐Thiol Exchange

**FIGURE 3:**
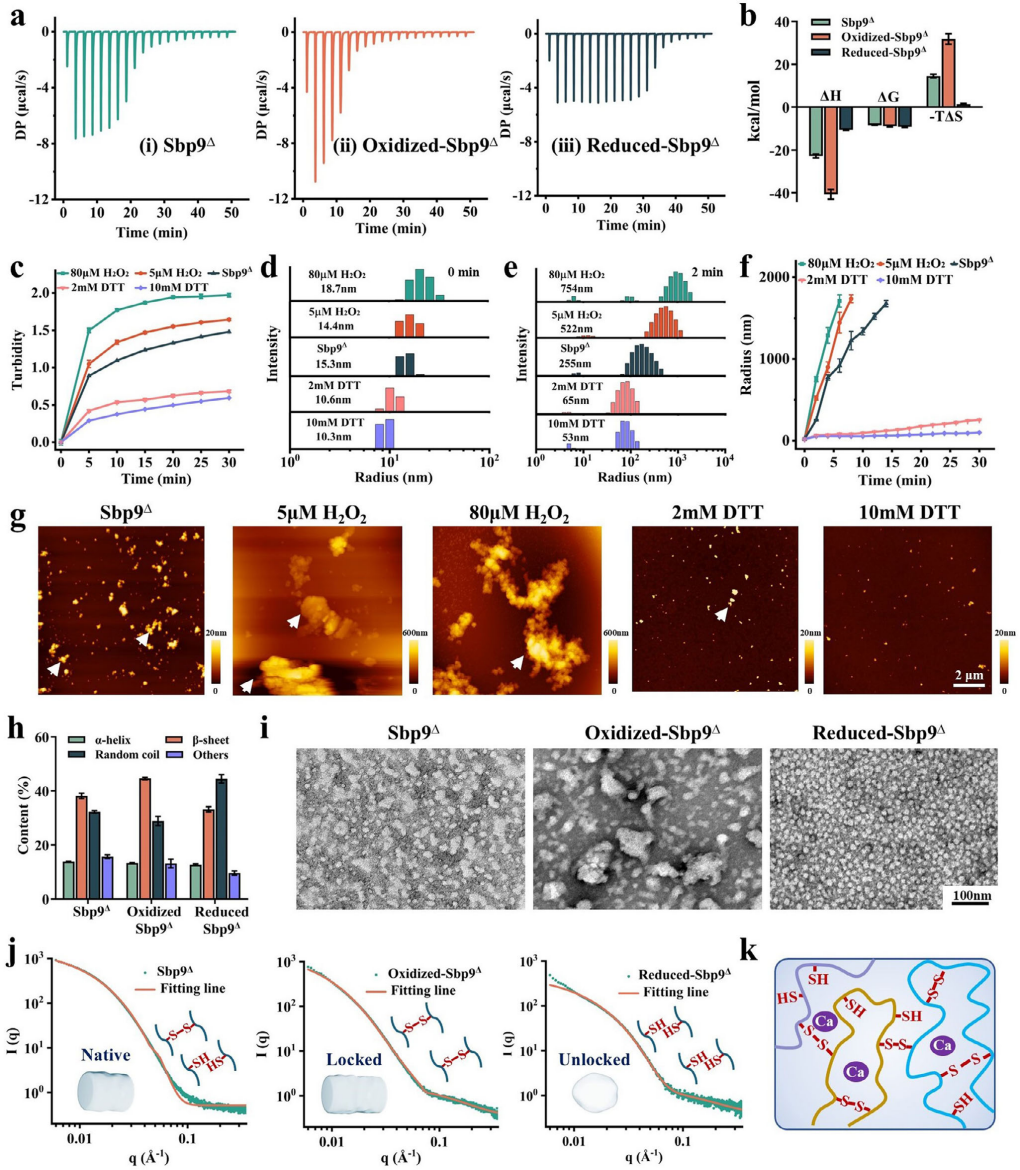
Redox‐dependent Sbp9^∆^ self‐assembly triggered by Ca^2+^. (a) Representative ITC thermograms for titrating 3 mM Ca^2+^ into 1.5 mg/mL Sbp9^∆^ under different redox conditions. Oxidized and reduced samples were pretreated with 5 µM H_2_O_2_ and 2 mM TCEP, respectively. (b) Corresponding thermodynamic parameters (Δ*H*, Δ*G*, and TΔ*S*). (c) Time‐dependent turbidity changes of Sbp9^∆^ solutions over 30 min. (d) Initial R_h_ and (e) R_h_ after 2 min of self‐assembly in the presence of varying H_2_O_2_ or DTT concentrations. (f) R_h_ changes of Sbp9^∆^ solutions under redox conditions over 30 min. (g) AFM images of protein aggregates at 2 min. (h) Secondary structure fractions of initial Sbp9^∆^, oxidized‐Sbp9^∆^, and reduced‐Sbp9^∆^, calculated from the amide I band of FTIR spectra. (i) TEM images of initial Sbp9^∆^ under different redox conditions. (j) SAXS profiles of the Sbp9^∆^, Oxidized‐Sbp9^∆^, and Reduced‐Sbp9^∆^. (k) Schematic illustration of Sbp9^∆^ self‐assembly under dual coordination by Ca^2+^ and disulfide bonds.

Given the redox‐sensitive modulation of coating formation, we further investigated the intrinsic self‐assembly properties of Sbp9^∆^, with a particular focus on its thermodynamic driving forces and aggregation behavior. Isothermal titration calorimetry (ITC) was employed to quantify the interactions between Ca^2+^ and Sbp9^∆^ [40]. Representative heat flow‐time curves are shown in Figure 3a. For native Sbp9^∆^, exothermic peaks gradually diminished and stabilized after the tenth injection. H_2_O_2_ treatment enhanced the binding intensity, with stabilization occurring after the sixth injection, whereas the reducing agent markedly weakened the binding interactions. Binding enthalpy (Δ*H*) and entropy term (TΔ*S*) are summarized in Figure 3b. The measured Δ*H* values were ‐22.9 kJ/mol for native Sbp9^∆^, −40.7 kJ/mol for oxidized‐Sbp9^∆^ and ‐10.6 kJ/mol for reduced‐Sbp9^∆^, indicating that the formation of disulfide bonds promotes a more exothermic Ca^2+^‐binding process. Correspondingly, the more negative TΔ*S* value of oxidized‐Sbp9^∆^ suggests conformational rigidification. ITC data reveal that the redox state of Sbp9^∆^ governs a trade‐off between binding strength and structural flexibility. The oxidized state provides a strong enthalpic driving force but a high entropic penalty, favoring rigid networks [41]. The reduced state exhibits weaker binding but greater flexibility. Native Sbp9^∆^, with its natural mix of free thiols and disulfide bonds, occupies an intermediate thermodynamic state. Notably, consistent with the redox responsiveness described above, the assembly of Sbp9^∆^, which is mediated by ionic coordination and disulfide crosslinking, is enthalpy‐driven, whereas the aggregation of amyloid‐like proteins, governed by hydrophobic interactions between adjacent chains, is entropy‐driven [42]. This fundamental distinction highlights how different proteins adopt tailored thermodynamic strategies to attain effective interfacial adhesion.

We further investigated redox‐sensitive assembly kinetics. H_2_O_2_ accelerated the nucleation and growth rate, with the 80 µM group reaching a turbidity of 1.9 at 30 min, compared to only 0.6 for the 10 mM DTT group (Figure 3c). Meanwhile, an increase in oxidant concentration raised R_h_ from 15.3 to 18.7 nm, whereas DTT dispersed aggregates, lowering R_h_ to approximately 10 nm (Figure 3d). Within 2 min of Ca^2+^ addition, the R_h_ of native Sbp9^∆^ rapidly rose to 255 nm (Figure 3e). The R_h_ values of the 5 and 80 µM groups increased sharply to 522 and 754 nm, respectively, whereas those of the 2 and 10 mM DTT groups showed only marginal increases, reaching 65 and 53 nm. Even after 30 min, DTT‐treated samples exhibited no significant increase in particle size (Figure 3f). Furthermore, AFM images demonstrate that native Sbp9^∆^ formed uniform aggregates, H_2_O_2_ promoted micron‐sized clusters, and DTT prevented aggregation (Figure 3g). Collectively, these results demonstrate that H_2_O_2_ accelerates Ca^2+^‐triggered self‐assembly by promoting intra‐ and intermolecular disulfide bond formation, whereas DTT suppresses nucleation and growth through the reduction of intrinsic disulfide bonds.

Although oxidation promotes rapid aggregate formation, the accompanying conformational rigidification may compromise assembly stability. In contrast, native Sbp9^∆^ achieves an optimal balance between binding strength and conformational flexibility, thereby enabling orderly and robust self‐assembly. This intermediate thermodynamic and kinetic profile likely represents an evolutionary adaptation to physiological redox fluctuations. Central to this adaptive behavior is the dynamic equilibrium of thiol‐disulfide exchange. Excessive oxidation prematurely locks the protein into kinetically trapped conformations, whereas the native state occupies a finely tuned regime that reconciles rapid assembly kinetics with thermodynamic stability.

To further evaluate whether the redox‐sensitive self‐assembly of Sbp9^Δ^ operates under physiologically relevant conditions, two biocompatible model systems were established. Glucose (Glu) and glucose oxidase (GOx) were added to Sbp9^Δ^ to generate a low but sustained oxidative flux, whereas glutathione (GSH) was employed to mimic the endogenous reducing environment [43]. The Glu/GOx system markedly increased solution turbidity, indicating accelerated assembly, whereas GSH treatment suppressed turbidity and prevented the formation of a dense network coating (Figure S5). These findings demonstrate that Sbp9^Δ^ self‐assembly is highly responsive to physiologically relevant redox gradients, further supporting the translational potential of this protein for on‐demand and microenvironment‐responsive adhesion in biomedical applications.

### 2.4 Disulfide Sticker‐Regulated Conformational Heterogeneity

Our macroscopic investigations demonstrated that Sbp9^∆^ self‐assembly is jointly regulated by Ca^2+^ and disulfide bonds, with Ca^2+^ serving as an initiator and disulfide bonds acting as dynamic stickers that stabilize the assembled network and confer interfacial adhesion. However, the nanoscale conformational heterogeneity underlying this process remains poorly understood. To address this gap, we combined spectroscopic and scattering techniques to elucidate how disulfide bonding influences the initial conformation and structural landscape prior to Ca^2+^‐activated assembly.

Previous studies have indicated that native Sbp9^∆^ exists in a highly polymerized state [21]. To probe redox‐dependent conformational changes, Fourier transform infrared spectroscopy (FTIR) was employed to analyze the secondary structure. Quantitative analysis of the amide  I band using Fourier self‐deconvolution and Gaussian fitting revealed that, relative to native Sbp9^∆^, additional disulfide bond formation increased the β‐sheet content from 38% to 45% while reducing the random‐coil content from 32% to 29% (Figure 3h). Conversely, disulfide bond cleavage led to a pronounced increase in random‑coil content. This redox‐driven conformational heterogeneity was further visualized by transmission electron microscopy (TEM) (Figure 3i). A striking morphological contrast was observed, where oxidized‐Sbp9^Δ^ predominantly formed large, irregular aggregates, consistent with extensive polymerization, whereas reduced‐Sbp9^Δ^ exhibited a more monodisperse morphology. Native Sbp9^Δ^ occupied an intermediate position along this morphological continuum, visually corroborating a moderate degree of polymerization.

Moreover, small‐angle X‐ray scattering (SAXS) provided direct, quantitative insight into the redox‐dependent conformational states of Sbp9^Δ^ at the nanoscale, a level of spatial resolution not accessible through ensemble‐averaged spectroscopic techniques alone [39]. As shown in Figure 3j, the SAXS profiles revealed pronounced differences in the radius of gyration (*R_g_*) between the oxidized and reduced forms. The data were further fitted using a cylindrical model to extract overall particle dimensions, quantified in terms of molecular length and width. Oxidized‐Sbp9^Δ^ exhibited a larger *R_g_* and more elongated dimensions, consistent with a locked conformation stabilized by inter‐ and intramolecular disulfide crosslinks (Table S1). This rigid, rod‑like architecture promotes spatial extension and facilitates efficient assembly into coatings. In contrast, reduced‐Sbp9^Δ^, lacking stabilizing disulfide bonds, adopted a more flexible, unlocked state with relaxed topological constraints. This inability to form stable intermolecular crosslinks was directly reflected in the SAXS data as a decreased *R_g_* and shortened molecular length. Notably, given the heterogeneity in particle size, polydispersity was assessed by fitting the scattering data with a Gaussian distribution. Collectively, these complementary morphological and structural parameters indicate that the dynamic formation and cleavage of disulfide bonds exert quantitative control over the assembly pathway of Sbp9^Δ^, from flexible individual chains to rigid supramolecular networks.

Differential scanning calorimetry (DSC) provided additional evidence for the conformational heterogeneity of Sbp9^∆^. Oxidized‐Sbp9^∆^ exhibited a higher melting temperature (*T*m) of 70.2°C, reflecting enhanced thermal stability and a greater degree of polymerization (Figure S6). By comparison, reduction of disulfide bonds lowered the *T*m to 38.6°C, consistent with a substantial loss of conformational stability [44]. Interestingly, a second endothermic peak emerged at 79.1°C, suggesting that even after overall depolymerization induced by disulfide cleavage, some dissociated components remain thermally stable. Taken together, these findings demonstrate that dynamic disulfide bonds act as critical regulators of the enthalpy‐driven, multiscale assembly of Sbp9^∆^ (Figure 3k). At the macroscale, disulfide crosslinking functions as a redox‐sensitive switch that governs both coating deposition and interfacial adhesion. This redox‐dependent tunability originates from conformational control at the nanoscale. Specifically, disulfide bond formation rigidifies the protein backbone, elevates β‐sheet content, and stabilizes an extended, rod‐like conformation. Conversely, reductive cleavage of disulfide bonds yields a flexible, coil‐rich conformation that is incompetent for productive assembly.

This redox plasticity is reconciled with the structural conservation of EGF‐like domains by invoking two functionally distinct populations of cysteine residues. Canonical cysteines form conserved intra‐domain disulfide bonds, providing a stable scaffold responsible for the residual thermal stability observed even in the reduced state (Figure S6). Superimposed on this conserved framework are surface‐exposed cysteines that engage in dynamic inter‐domain and intermolecular disulfide exchange. The presence of free thiols in the native protein and the dramatic redox‐switchable assembly indicate the formation of an intermolecular disulfide network upon oxidation. Consequently, the observed redox plasticity does not disrupt the fundamental EGF‐like fold but instead represents a tunable interaction layer that endows Sbp9^∆^ protein with its functional sticker properties.

Notably, the disulfide bond functions as a molecular lock, and Ca^2+^ acts as a kinetic trigger, operating in a non‑mutually exclusive manner on distinct timescales and hierarchical levels. Disulfide bonds predominate in defining the building block and conferring cohesive stability to the network. Their role is to establish a state of conformational preparedness and to provide durable intermolecular crosslinks that sustain the assembled architecture. In contrast, Ca^2+^ predominates in initiating assembly and in dictating network topology. Furthermore, the disulfide‐stabilized conformation provides the structural foundation for cooperative Ca^2+^ binding at the nanoscale. Disulfide bonds create a rigid, pre‐organized scaffold that amplifies the enthalpic gain of Ca^2+^ coordination while simultaneously shifting the primary entropy penalty from the protein backbone to the solvent, thereby facilitating the formation of a thermodynamically stable network.

### 2.5 Robust Wet Adhesion and Notable Antioxidant Activity of Sbp9^∆^ Coatings

**FIGURE 4:**
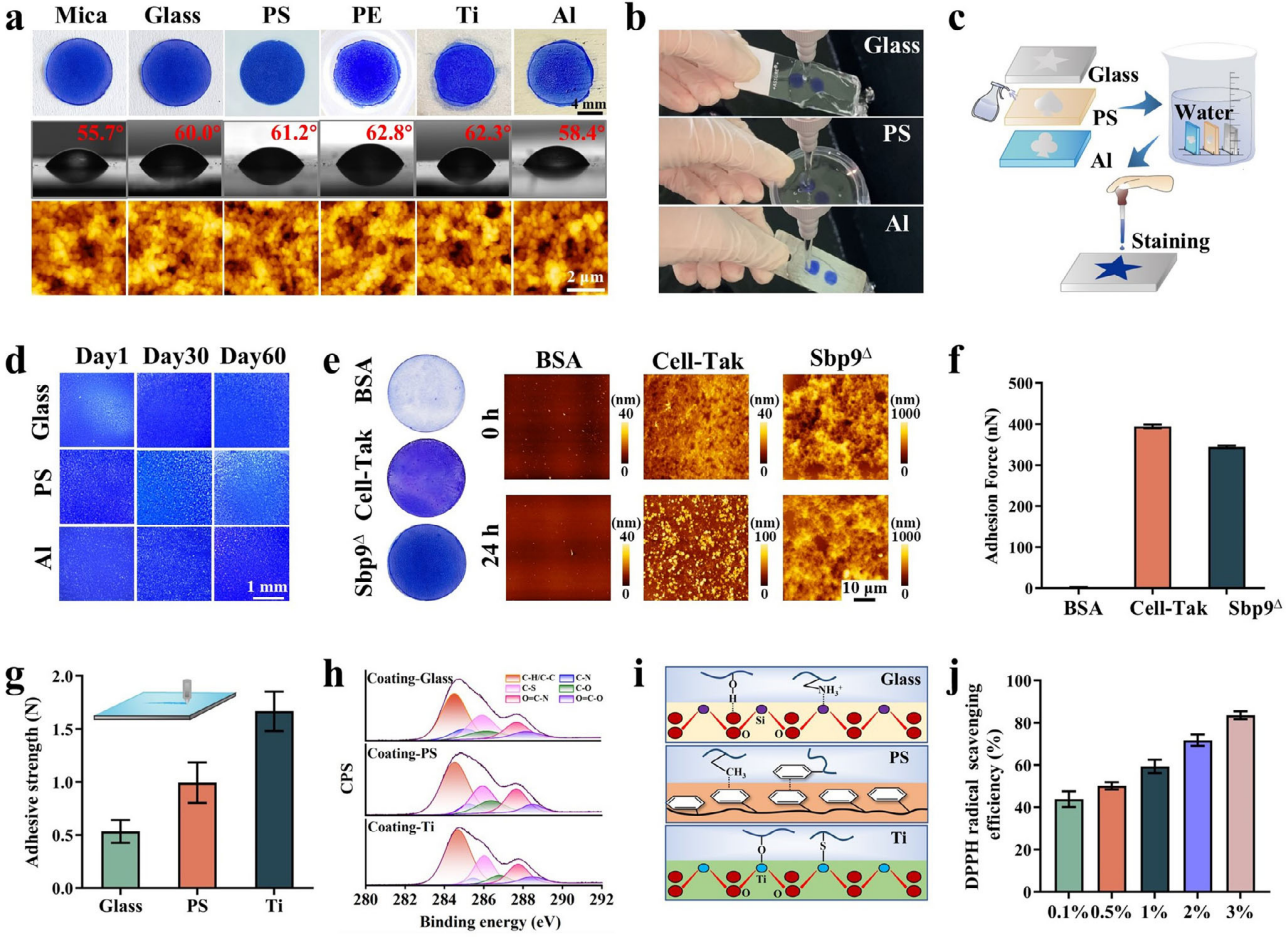
Characterization of Sbp9^∆^ coatings in terms of wet stability, nanomechanical properties, universal adsorption, and antioxidant activity. (a) Water contact angle (WCA) and AFM images of Sbp9^∆^ coatings deposited on various substrates including polystyrene (PS), polyethylene (PE), titanium (Ti), and aluminum (Al). (b) Photographs of PS, Al, and glass substrates coated with Sbp9^∆^ after rinsing under running tap water. (c) Schematic of the wet stability test, in which coatings on glass, PS, and Al were exposed to flowing water for 60 days. (d) Analysis of coating integrity at days 1, 30, and 60. (e) Morphological comparison of BSA, Cell‐Tak, and Sbp9^∆^ coatings after 24 h of agitation under flowing water. Scale bar = 10 µm. (f) Adhesion force of the protein coatings on silicon substrates measured by AFM. (g) Adhesive strength of Sbp9^∆^ coatings on glass, PS, and Ti assessed via nano‐scratch testing. (h) High‐resolution C1s XPS spectra of Sbp9^∆^ coatings on glass, PS, and Ti. (i) Schematic illustrating proposed interactions between Sbp9^∆^ coatings and substrate surfaces. (j) DPPH radical scavenging activity as a function of protein concentration.

To evaluate the Sbp9^∆^ coating performance under application‐relevant conditions, the adsorption capacity, wet stability, and mechanical properties were characterized. Substrates including mica, glass, polystyrene (PS), polyethylene (PE), titanium (Ti), and aluminum (Al) exhibited consistent water contact angles (55.7°–62.8°) and uniform nanoparticle‐based morphologies, confirming successful coating deposition across diverse substrates (Figure 4a and Figure S7). Wet stability was examined under harsh conditions, including flushing, ultrasonic treatment, and immersion in flowing water. Coatings on glass, PS, and Al remained largely intact after 30 min of water flushing and 1 h of ultrasonic destruction (Figure 4b and Movie S1). Moreover, the coatings preserved mesh structural integrity following 60‐day water immersion (Figure 4c,d), with an area retention rate exceeding 88% (Figure S8). These results indicate superior stability in humid environments, meeting a key requirement for in situ tissue engineering applications. For comparison, coatings of Cell‐Tak (a commercially available product consisting of mixtures of naturally extracted mussel foot proteins) and BSA were prepared on glass slides [45]. The wet stability of these coatings was first evaluated (Figure 4e). In contrast to the porous network of the Sbp9^∆^ coating, Cell‐Tak initially formed a continuous, non‐porous film. After 24 h in flowing water, however, this film fragmented into scattered spherical nanoparticles, whereas the Sbp9^∆^ coating retained its original microstructure, highlighting its exceptional wet stability and adhesion. Interfacial adhesion was further quantified via AFM [46]. While the BSA coating exhibited negligible adhesion, the adhesion forces for Cell‐Tak and Sbp9^∆^ coatings were 394 nN and 345 nN, respectively, confirming the substantial adhesive capability of the recombinant scallop protein (Figure 4f and Figure S9).

Nano‐scratch testing revealed robust interfacial adhesion of Sbp9^∆^ coating across multiple substrates, with the highest adhesion strength of 1.67 N on titanium (Ti) (Figure 4g and Figure S10). High‐resolution XPS C1s spectra further revealed the presence of diverse functional groups on the coating surface, which likely contribute to its universal adsorption (Figure 4h and Figure S11). These findings suggest that adhesion to glass is primarily mediated by hydrogen bonds or electrostatic interactions, adhesion to PS is driven predominantly by hydrophobic interactions, and adhesion to Ti involves coordination bonding (Figure 4i) [47]. Meanwhile, the in vitro antioxidant assays demonstrated concentration‐dependent activity, with DPPH radical scavenging rates increasing from 44% to 84% as the protein concentration rose from 0.1% to 3%, indicating enhanced antioxidant capacity at higher concentrations (Figure 4j). Collectively, the combination of reliable adsorption capability, sustained wet stability, and tunable antioxidant activity makes the Sbp9^∆^ coating a highly versatile and promising platform for broad biomedical applications.

### 2.6 Evaluation of Sbp9^∆^ Coatings for Hair Regrowth

**FIGURE 5:**
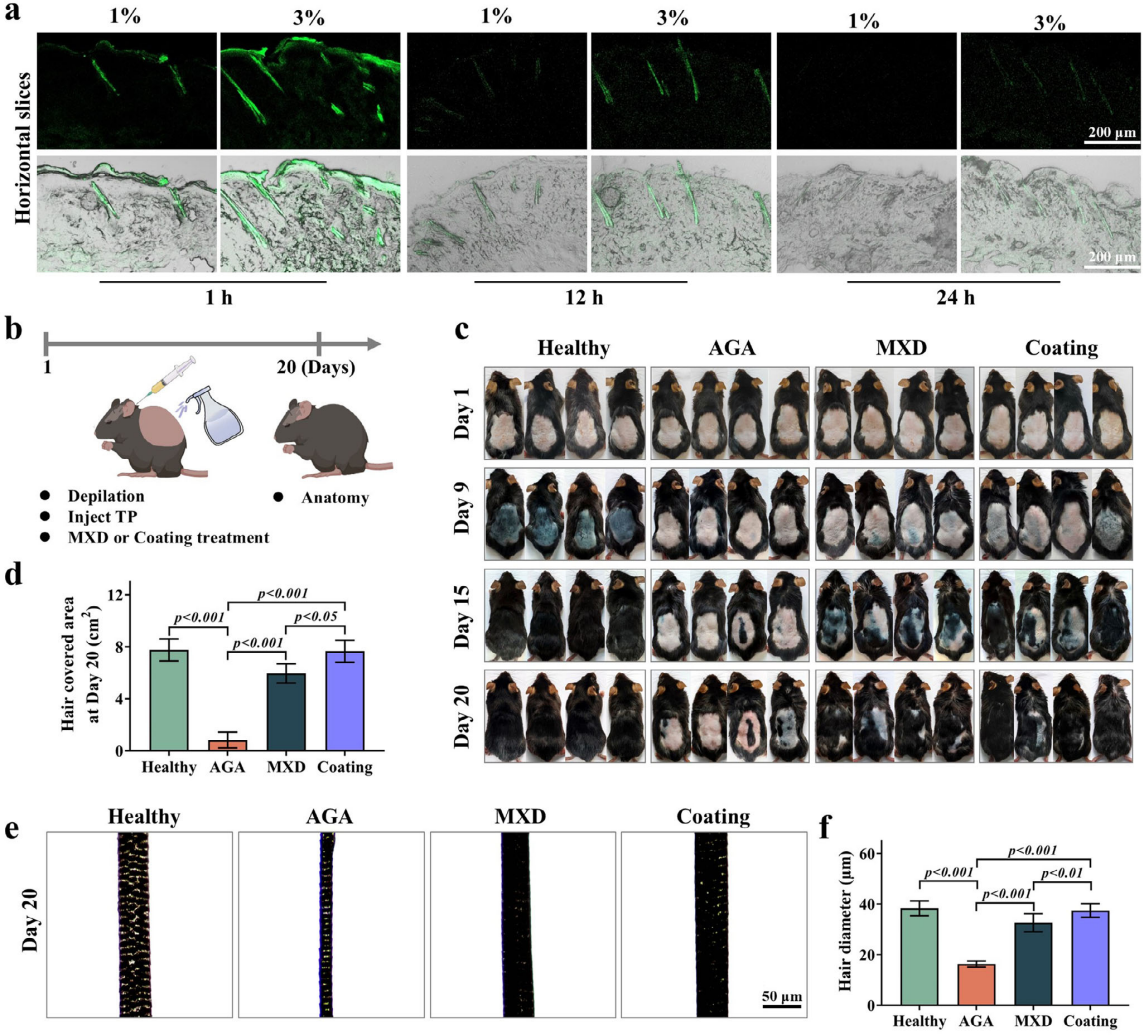
Sbp9^∆^ coating promotes hair regrowth in an AGA mouse model. (a) Representative fluorescence microscopy images of horizontal dorsal skin treated with 1% and 3% FITC‐Sbp9^∆^ coatings at 1, 12, and 24 h post‐administration. (b) Schematic of AGA mouse model establishment and treatment strategies. TP, testosterone propionate. MXD, minoxidil. (c) Representative photographs of hair regrowth in healthy, AGA, MXD, and coating groups. (d) Quantified hair‐covered area on dorsal skin at day 20 post‐depilation (*n* = 4). (e) Images of regenerated hair at day 20 post‐depilation. Scale bar = 50 µm. (f) Diameter of regenerated hair at day 20 post‐depilation (*n* = 10).

The Sbp9^∆^ coating demonstrated a compelling profile, featuring a facile one‐step in situ fabrication process, robust wet adhesion, and outstanding antioxidant activity attributed to abundant Cys residues. Furthermore, the biocompatible coating has been shown to enhance fibroblast and endothelial cell migration and proliferation, thereby accelerating diabetic wound healing [21]. Based on these properties, we explored the application of the Sbp9^∆^ coatings in promoting hair regeneration. To evaluate penetration, 1% and 3% coatings were applied to mouse skin, which was sectioned both longitudinally and horizontally for fluorescence monitoring over a 24 h period. The coating penetrated the epidermis and reached the perifollicular dermis within 1 h, with fluorescence intensity decreasing markedly at 12 and 24 h (Figure S12). The significantly higher fluorescence intensity observed with the 3% coating indicated that higher protein concentration facilitated enrichment of active ingredients. Horizontal sections further confirmed that 3% coating achieved deeper and stronger penetration, providing a rationale for selecting the dosing concentration used in the in vivo hair regeneration study (Figure 5a and Figure S13).

AGA, the most prevalent form of hair loss, is closely linked to redox imbalance [10, 48]. To assess the therapeutic potential of the Sbp9^∆^ coating, an AGA model was established via testosterone injection (Figure 5b) [49]. Commercial minoxidil tincture, the Food and Drug Administration (FDA)‐approved topical treatment for hair loss, served as the positive control [6]. During the telogen‐to‐anagen transition, follicular melanin secretion induces skin pigmentation, causing a visual shift from pink to darker skin [50]. Representative images of hair regrowth across different groups at various time points are presented in Figure 5c. On day 9, no hair regrowth was observed in the AGA group, whereas healthy mice had already initiated new hair growth, confirming successful model establishment. At this time point, the coating group exhibited more extensive skin pigmentation than the minoxidil group. On day 15, mice in both the minoxidil and coating groups showed visible hairs, with substantially greater hair coverage observed by day 20 following depilation. These observations preliminarily suggest that the Sbp9^∆^ coating possesses hair‐promoting efficacy comparable to that of minoxidil.

A systematic quantitative evaluation of hair coverage area, hair diameter, and follicle number was conducted to further assess regenerative potential [4]. On day 20, the minoxidil group achieved a hair coverage area of 5.96 cm^2^, whereas the coating group reached 7.65 cm^2^, indicating nearly complete coverage of the alopecic region by new hair (Figure 5d). The Sbp9^∆^ coating also promoted superior hair morphology relative to minoxidil (Figure 5e). Specifically, newly regenerated hairs in the coating group displayed intact cuticular scales and a greater mean diameter (37.44 µm) compared to those in the minoxidil group (32.64 µm). In contrast, hairs in the AGA group were significantly finer (16.31 µm) (Figure 5f). These findings indicate that the Sbp9^∆^ coating promotes hair regeneration more effectively than minoxidil, as reflected by enhanced outcomes in hair coverage, hair diameter, and follicular density.

**FIGURE 6:**
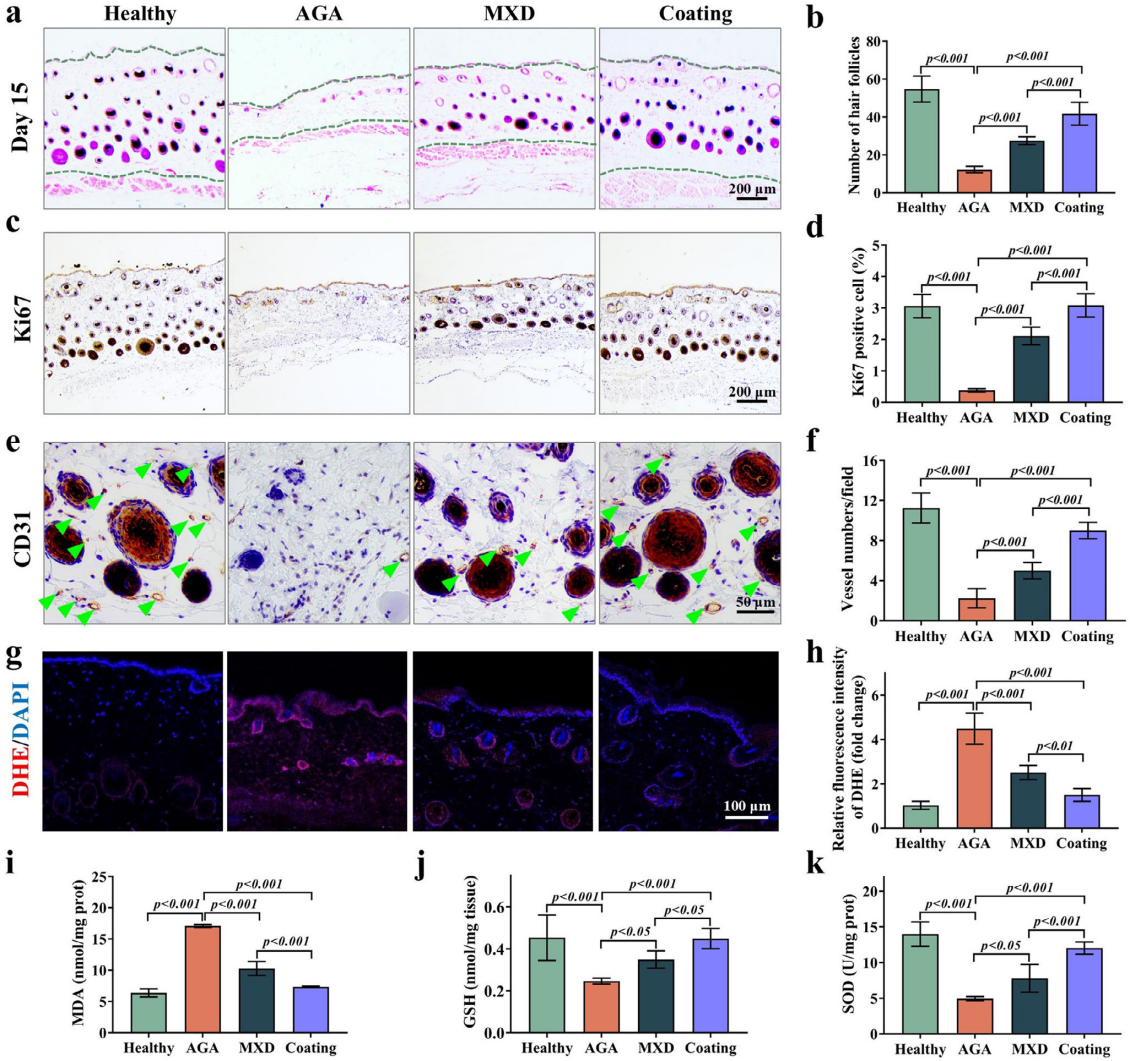
Sbp9^∆^ coating promotes hair regeneration by reshaping the perifollicular microenvironment. (a) Hematoxylin and eosin (H&E) staining of skin sections at day 15 post‐depilation. Green dashed lines delineate the regions containing hair follicles used for skin thickness measurements. Scale bar = 200 µm. (b) Quantification of hair follicle number per field of view at day 15 post‐depilation. (c) Representative immunohistochemistry images of Ki67 at day 15 post‐depilation. Scale bar = 200 µm. (d) Quantification of Ki67‐positive cells (*n* = 4). (e) Representative immunohistochemistry images of CD31 at day 15 post‐depilation. Arrows indicate blood vessels. Scale bar = 50 µm. (f) Quantification of blood vessel number per field of view (*n* = 4). (g) Representative confocal fluorescence images of DHE staining in skin tissues. Red, DHE. Blue, DAPI. Scale bar = 100 µm. (h) Quantitative analysis of DHE fluorescence intensity (*n* = 4). Levels of (i) malondialdehyde (MDA), (j) glutathione (GSH), and (k) superoxide dismutase (SOD) in the dorsal skin tissues (*n* = 4).

Histological analysis of skin tissues after 15 days of treatment provided further insight into the regenerative effects of the Sbp9^∆^ coating. H&E staining revealed only sparse hair follicles in the AGA group, whereas both treatment groups exhibited a high density of enlarged hair bulbs within the subcutaneous tissue (Figure 6a). Notably, the coating group displayed the most active follicular morphology, indicative of an accelerated telogen‐to‐anagen transition (Figure 6b) [51]. Skin thickness in the coating group was also significantly greater than that in both the AGA and minoxidil groups on day 15, demonstrating that the coating effectively mitigated testosterone‐induced follicular damage (Figure S14). Meanwhile, immunohistochemical staining was performed to evaluate cell proliferation and angiogenesis in the vicinity of newly formed hair follicles. As previously reported, induction of the anagen phase is closely associated with rapid cellular proliferation, including that of dermal papilla cells and hair follicle stem cells [48]. Accordingly, the expression of Ki67, a canonical proliferation marker, was examined. As shown in Figure 6c,d, only sparse Ki67‐positive signals were detected in the AGA group on day 15, whereas the coating group exhibited a marked increase in Ki67 expression, indicating enhanced proliferation of germinal matrix cells. Given that an adequate vascular supply is essential for the telogen‐to‐anagen transition, angiogenesis was further evaluated by CD31 immunostaining [52]. The coating group exhibited the highest density of perifollicular blood vessels among all groups, surpassing both the AGA and minoxidil groups. These findings confirm its superior pro‐angiogenic activity and its ability to modulate the local follicular microenvironment (Figure 6e,f).

Additionally, the pathological environment of AGA is characterized by elevated ROS levels, which inhibit follicular activation [53]. Given the established antioxidant capacity of the Sbp9^∆^ coating, we hypothesized that it could mitigate perifollicular oxidative stress [21]. DHE staining of tissue sections revealed substantially lower fluorescence in the coating group (Figure 6g). Quantitative analysis showed a 4.46‐fold increase in DHE intensity in the AGA group relative to healthy controls, whereas the minoxidil and coating groups showed only 2.49‐ and 1.46‐fold increases, respectively (Figure 6h). These results highlight the superior capacity of the Sbp9^∆^ coating to eliminate pathological ROS and alleviate oxidative suppression of hair growth [1]. To further evaluate in vivo antioxidant efficacy, levels of malondialdehyde (MDA), glutathione (GSH), and superoxide dismutase (SOD) were measured in day 15 skin tissues [15]. As illustrated in Figure 6i, the pronounced MDA elevation in the AGA group was significantly attenuated by both minoxidil and coating treatments. Remarkably, the coating group restored MDA to a level comparable to that of the healthy group, confirming effective suppression of lipid peroxidation. Concurrently, the coating group exhibited a substantial increase in GSH levels and a significant enhancement of SOD activity, collectively rebalancing the redox environment to favor follicle growth (Figure 6j,k). Taken together, the Sbp9^∆^ coating accelerates cell proliferation and angiogenesis while alleviating oxidative stress through ROS scavenging, enhancement of antioxidant capacity, and restoration of redox homeostasis.

Finally, as biocompatibility is essential for biomedical translation, systemic safety of the Sbp9^∆^ coating was evaluated. A hemolysis assay showed no significant hemolysis, confirming compatibility with red blood cells (Figure S15a). In a 7‐day skin sensitivity test on SD rats, no erythema or edema was observed, and the coating was classified as nonirritating according to established criteria (Table S2). H&E staining of treated skin revealed no foreign body invasion or inflammatory cell infiltration, with inflammatory cells identified by their characteristic large nuclei and high nucleocytoplasmic ratio (Figure S15b). Furthermore, histological examination of major organs (heart, liver, spleen, lung, kidney) revealed no signs of inflammation or damage (Figure S16). Collectively, these results demonstrate the excellent biosafety and biocompatibility of the Sbp9^∆^ coating.

### 2.7 Wnt and Calcium Signaling Pathways Reveal Coating‐Induced Hair Regeneration

**FIGURE 7:**
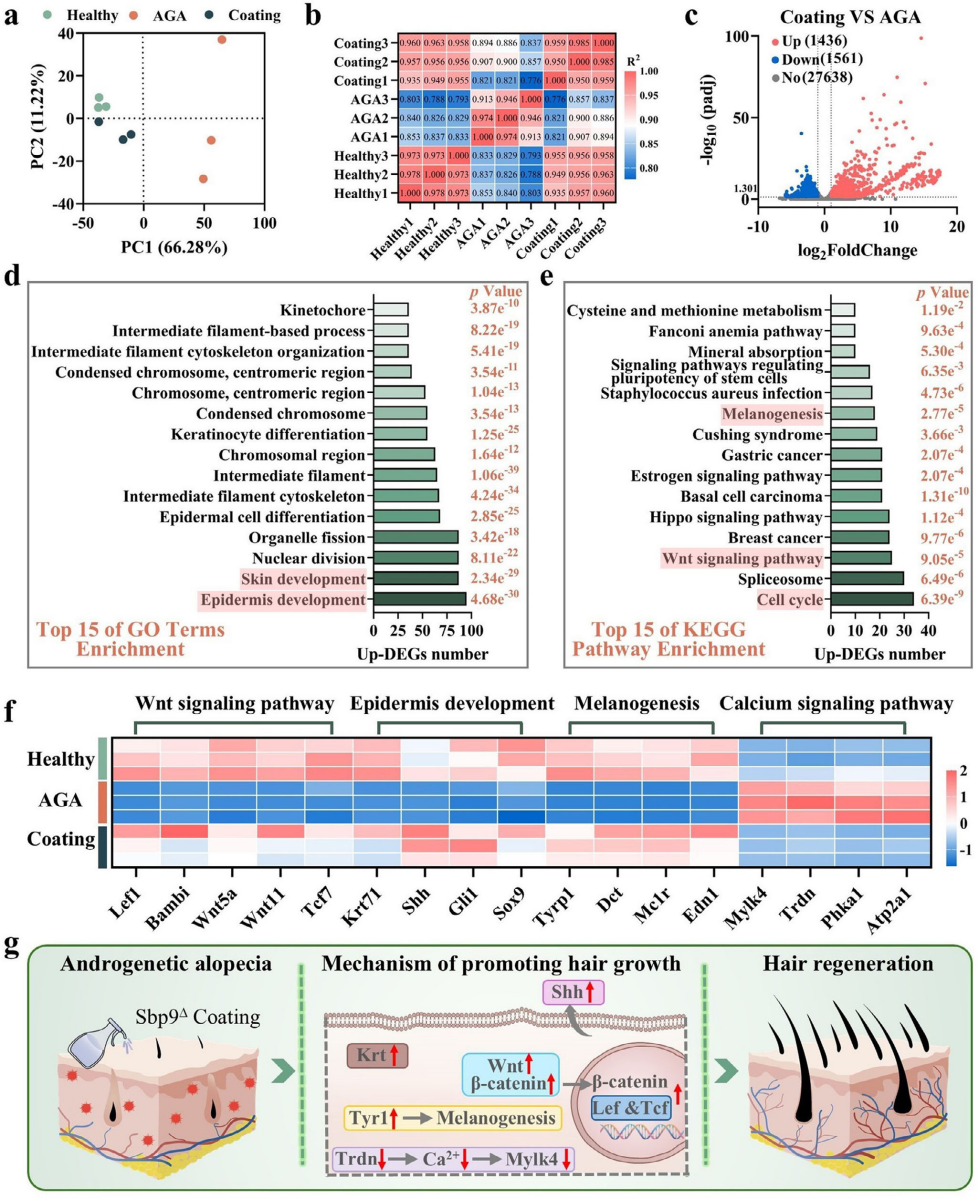
Transcriptomic analysis of the molecular mechanisms underlying Sbp9^∆^ coating‐induced hair regeneration. (a) Principal component analysis (PCA) of gene expression profiles. (b) Pearson correlation analysis of transcriptomic data revealing high intra‐group consistency and pronounced inter‐group divergence. (c) Volcano plot of differentially expressed genes (DEGs) between the AGA group and the Sbp9^∆^ coating group. (d) Gene Ontology (GO) pathway enrichment analysis and (e) Kyoto Encyclopedia of Genes and Genomes (KEGG) pathway enrichment analysis, showing the top enriched pathways upregulated in the coating group relative to the AGA group. (f) Heatmap displaying expression patterns of representative genes associated with significantly enriched pathways. (g) Schematic illustration summarizing the proposed mechanism by which the Sbp9^∆^ coating promotes hair regeneration in androgenetic alopecia.

To gain comprehensive insight into the molecular mechanisms underlying Sbp9^∆^ coating‐mediated hair regeneration, transcriptomic analysis was performed on dorsal skin tissues from AGA mice. Principal component analysis (PCA) distinctly separated the coating and AGA groups into discrete clusters, indicating substantial transcriptomic reprogramming (Figure 7a). The Pearson correlation heatmap revealed high consistency among biological replicates within each group, confirming the robustness and reproducibility of the dataset (Figure 7b). As shown in the volcano plot, 2997 differentially expressed genes (DEGs) were identified in the coating group relative to the AGA group, comprising 1436 upregulated and 1561 downregulated genes, reflecting the transcriptional changes underlying the observed enhancement in hair regeneration (Figure 7c).

Gene Ontology (GO) enrichment analysis of the DEGs revealed significant modulation of biological processes critical to follicular function. Upregulated biological processes included pathways associated with hair follicle regeneration, such as epidermal development, keratinization, and intermediate filament cytoskeleton organization (Figure 7d). Conversely, the Sbp9^∆^ coating potently suppressed pathogenic processes, including muscle cell differentiation, which has been associated with follicular miniaturization and prolonged telogen (Figure S17a). To further investigate the underlying cellular signaling mechanisms, Kyoto Encyclopedia of Genes and Genomes (KEGG) pathway analysis was performed. The Sbp9^∆^ coating significantly upregulated key pathways implicated in hair follicle development and regeneration, including the cell cycle, Wnt signaling, Hippo signaling, and melanogenesis pathways (Figure 7e). Among these, Wnt signaling is critically required for dermal papilla cell activation, anagen induction, and stem cell fate determination. Additionally, enrichment of the melanogenesis pathway suggests that the coating may actively promote melanocyte‐mediated hair pigmentation [54]. Dihydrotestosterone has been reported to induce calcium influx, thereby triggering excessive oxidative stress and apoptosis in a calcium signaling‐dependent manner and promoting the transition of hair follicles from anagen to catagen [55, 56]. Notably, the Sbp9^∆^ coating downregulated the calcium signaling pathway, thereby suppressing follicular regression and partially reversing the pathological microenvironment associated with AGA (Figure S17b).

We next examined the DEGs enriched in the Wnt signaling pathway, epidermal development, melanogenesis, and calcium signaling pathway (Figure 7f). Specifically, the heatmap of DEGs in the Wnt signaling pathway demonstrated elevated expression of *Lef1*, *Bambi*, *Wnt5a*, *Wnt11*, and *Tcf7* in both the coating and healthy groups. In parallel, heatmap visualization of calcium signaling components showed broad downregulation of genes including *Mylk4*, *Trdn*, *Phka1*, and *Atp2a1*. Hair growth‐regulatory genes were further validated by qRT‐PCR (Figure S18). Consistent with the transcriptomic data, the expression levels of *Lef1*, *Bambi*, *Wnt5a*, *Tcf7*, *Krt71*, *Shh*, *Tyrp1*, and *Dct* in the coating group were comparable to those in the healthy group and markedly higher than those in the AGA group. In contrast, the calcium signaling‐related genes *Mylk4* and *Trdn* were substantially downregulated in both the coating and healthy groups relative to the AGA group. Collectively, transcriptomic profiling revealed that the Sbp9^∆^ coating remodels the follicular microenvironment via coordinated proliferative activation and suppression of pathological processes (Figure 7g). These changes promote a robust and durable regenerative state, highlighting the Sbp9^∆^ coating as a promising and mechanistically grounded therapeutic strategy for AGA treatment.

Notably, the present Sbp9^∆^ coating system does not rely on conventional microneedles or permeation‑enhancing additives, representing a potentially novel class of regenerative material [3, 57]. Although large protein molecules are generally restricted by the stratum corneum, we propose that Sbp9^∆^ contains multiple active motifs capable of activating keratinocytes and melanocytes [21, 58]. This activation may stimulate growth factor secretion and initiate top‑down signaling to the dermis, ultimately modulating the perifollicular microenvironment [59]. Such indirect signaling likely necessitates higher topical protein concentrations, as evidenced by the concentration‑dependent antioxidant efficacy observed between 0.1% and 3% formulations. Furthermore, the coating forms a protective film on the skin surface that acts as a physical barrier against external stimuli and helps maintain localized humidity around follicular openings. The soft, proteinaceous matrix may also supply ECM‐like mechanical cues to the epidermis, further promoting follicular regeneration. Collectively, these microenvironmental contributions support follicular health and normal cycling [60].

## 3 Conclusion

In this study, we elucidate a disulfide‐sticker mechanism that underpins the hierarchical self‐assembly of marine adhesive proteins. Through a multi‐scale investigation of the marine adhesive protein Sbp9^Δ^, which contains EGF‐like repeats, we demonstrate that dynamic disulfide bonds operate synergistically with Ca^2+^ coordination to direct the formation of highly ordered nanostructures. Integrated biophysical analyses reveal that disulfide bonds serve as covalent locks, driving the rigidification of Sbp9^Δ^ into a β‐sheet‐rich, elongated rod‐like conformation. The Sbp9^Δ^ coating exhibits robust wet adsorption on diverse substrates and possesses intrinsic antioxidant activity conferred by its cysteine‐rich composition. In a biomedical application, the Sbp9^Δ^ coating significantly promotes hair regeneration in a model of androgenetic alopecia, outperforming minoxidil in terms of hair coverage, hair diameter, and follicular density. Mechanistically, the coating remodels the hostile perifollicular microenvironment by upregulating cell cycle, Wnt, Hippo, and melanogenesis pathways while downregulating calcium signaling pathway, thereby accelerating the telogen‐to‐anagen transition. These findings uncover a disulfide‐mediated assembly paradigm in marine bioadhesives that differs from conventional Dopa‐ and metal‐coordination‐centered models. Moreover, the generalizability of the disulfide‑sticker strategy to EGF‑like domains and other cysteine‑rich modular proteins requires further validation, owing to the extensive diversity in sequence architecture, structural organization, and environmental responsiveness.

## 4 Experimental Section

### 4.1 Protein Expression and Purification

The recombinant plasmid encoding Sbp9^Δ^ was inserted into a modified pET‐32a vector (pET‐32aHisTT) and transformed into *E. coli* BL21 (DE3) competent cells. Transformed cells were cultured in LB medium supplemented with 50 mg/L kanamycin at 37°C. Protein expression was induced with 0.2 mM isopropyl‐β‐D‐thiogalactopyranoside (IPTG) when the optical density at 600 nm reached approximately 0.6. The temperature was then lowered to 16°C, and cultivation was continued for 20 h. The cell pellet was solubilized with 1 mM urea, dissolved in 8 M urea buffer containing 10 mM DTT. Finally, the purified protein was refolded by dialysis against 20 mM Tris‐HCl buffer (pH 8.5).

### 4.2 Ca^2+^‐Induced Phase Separation

To induce phase separation, 50 µL of CaCl_2_ was added to 450 µL of 0.6 mg/mL Sbp9^Δ^ solution to achieve a final Ca^2+^ concentration of 5 mM. The mixture was immediately transferred to a glass vial. After incubation at room temperature for 30 min, the vial was inverted to observe separation between the solid and liquid phases. To confirm Ca^2+^‐induced phase separation behavior, 500 µL of Sbp9^Δ^ solution without Ca^2+^ was used for the control experiment and kept at room temperature. For redox‐mediated phase separation, the protein solutions were pre‐incubated with either 5 µM H_2_O_2_ or 2 mM DTT.

### 4.3 Coating Formation and Morphology Observation

For optical microscopy observation, a coating was formed by mixing 5 µL of CaCl_2_ solution with 45 µL of Sbp9^Δ^ solution. The mixture was immediately transferred onto a clean, round glass slide (1 cm in diameter) and incubated at 25°C for 30 min. For comparison, coatings of BSA (1 mg/mL) and Cell‐Tak (1.44 mg/mL) were also prepared by depositing 50 µL of each protein solution on glass slides for 12 h at 25°C. Subsequently, all slides were thoroughly washed with ultrapure water to remove non‐adsorbed proteins, stained with Coomassie Brilliant Blue, and imaged using a Motic AE2000 microscope.

For redox‐mediated phase separation, the protein solutions were pre‐incubated with 5 µM H_2_O_2_, 80 µM H_2_O_2_, 2 mM DTT, and 10 mM DTT, respectively. For AFM analysis, self‐assembly was initiated by mixing 45 µL of Sbp9^Δ^ solution (0.6 mg/mL) with 5 µL of 10 mM CaCl_2_. At designated time points during self‐assembly, 10 µL of the reaction mixture was withdrawn, deposited onto a freshly cleaved mica surface, and air‐dried. The morphology of the assembled structures was then examined using an AFM 5100N instrument (Hitachi, Japan) operated in tapping mode.

### 4.4 Turbidity Assay

The protein self‐assembly process was monitored by measuring changes in solution turbidity. Specifically, 20 µL of 10 mM CaCl_2_ was mixed with 180 µL of Sbp9^Δ^ solution (0.6 mg/mL), and the resulting mixture was immediately transferred to a 96‐well plate. Absorbance at 340 nm was recorded over a period of 30 min using a BioTek Synergy H1 microplate reader (Agilent, USA), with measurements taken at 5 min intervals. For the Sbp9^Δ^ self‐assembly in the absence of Ca^2+^, absorbance was monitored over a period of 40 h. For redox‐mediated self‐assembly, the protein solutions were pre‐incubated with 5 µM H_2_O_2_, 80 µM H_2_O_2_, 2 mM DTT, and 10 mM DTT, respectively. Each sample was measured in triplicate.

### 4.5 Dynamic Light Scattering (DLS)

The hydrodynamic size of Sbp9^Δ^ during self‐assembly was monitored using a DynaPro NanoStar instrument (Wyatt Technology, USA). To initiate self‐assembly, 5 µL of CaCl_2_ was mixed with 45 µL of Sbp9^Δ^ solution, and the mixture was immediately transferred to a capped quartz cuvette. Measurements were performed at 25°C. For the Ca^2+^‐triggered self‐assembly, data were collected continuously over 30 min. As a control, the size of Sbp9^Δ^ solution without Ca^2+^ addition was also monitored under the same conditions for 40 h. For redox‐mediated self‐assembly, the protein solutions were pre‐incubated with 5 µM H_2_O_2_, 80 µM H_2_O_2_, 2 mM DTT, and 10 mM DTT, respectively. Each sample was measured in triplicate.

### 4.6 Isothermal Titration Calorimetry (ITC)

The thermodynamic parameters of CaCl_2_ binding to Sbp9^Δ^ were determined using a MicroCal PEAQ‐ITC system (Malvern, UK). To evaluate the effect of redox environment, protein solutions were pre‐incubated with either 5 µM H_2_O_2_ or 2 mM TCEP. The sample cell was loaded with 300 µL of protein solution (1.5 mg/mL), and the syringe was filled with CaCl_2_ solution (3 mM). Titrations were performed at 25°C with a stirring speed of 750 rpm. Following an initial dummy injection of 0.4 µL (excluded from analysis), 19 successive injections of 1 µL were performed at intervals of 150 s. The heat of dilution, obtained from titrating CaCl_2_ into buffer alone, was subtracted from each titration curve. The data were fitted using a single‐site binding model to determine the enthalpy change (∆*H*) and entropy change (∆*S*). Each sample was measured in triplicate.

### 4.7 Differential Scanning Calorimetry (DSC)

The thermal stability was assessed using a MicroCal PEAQ‐DSC instrument (Malvern, UK). To evaluate the effect of redox environment, protein solutions were pre‐incubated with either 5 µM H_2_O_2_ or 2 mM Tris(2‐carboxyethyl)phosphine hydrochloride (TCEP). For each measurement, 250 µL of the protein solution was loaded into the sample cell, while an equivalent volume of 20 mM Tris‐HCl was loaded into the reference cell. The temperature was scanned from 25°C to 100°C at a rate of 60°C/h. After completing the scan, the buffer‐buffer baseline was subtracted from the sample thermogram. The resulting curve was fitted using a non‑two‑state model to determine the melting temperature (*Tm*). Each sample was measured in triplicate.

### 4.8 Fourier Transform Infrared Spectroscopy (FTIR)

The secondary structure was analyzed using an FTIR Spotlight 200i spectrometer (PerkinElmer, US). To evaluate the influence of redox environment, protein solutions were pre‐incubated with either 5 µM H_2_O_2_ or 2 mM TCEP. All spectra were recorded with a resolution of 2/cm, and the wavenumbers ranged from 500 to 4000/cm. Spectral analysis was performed using OMNIC 8.2 software, and the area under the amide I region (1600–1700/cm) was decomposed using PeakFit 4.12 software. The spectra were characterized by strong absorption at about 1610–1640/cm corresponding to β‐sheet, 1640–1648/cm assigned to random coil, and 1648–1660/cm assigned to α‐helix.

### 4.9 Transmission Electron Microscope (TEM)

The protein sample (10 µL) was applied onto a hydrophilized 200‐mesh copper grid and incubated for 2 min. Excess sample was removed using filter paper held perpendicular to the grid. Following 3 min of air‐drying, the grid was stained with 10 µL of uranyl acetate for 2 min, and air‐dried for an additional 3 min. TEM imaging was conducted on a JEM‐1400Flash microscope (JEOL, Japan) operated at an accelerating voltage of 120 kV.

### 4.10 N‐(1‐Pyrenyl)Maleimide (NPM) Staining

The formation and reduction of disulfide bonds in Sbp9^Δ^ were monitored using the thiol‑sensitive fluorescent probe NPM. Briefly, 1 µL of 10 mM NPM (in DMSO) was mixed with 200 µL of protein solution (0.1 mg/mL) in a 96‐well plate at room temperature. Fluorescence intensity was measured on a BioTek Synergy H1 microplate reader (Agilent, USA) with excitation and emission wavelengths of 330 and 375 nm, respectively. Each sample was measured in triplicate.

### 4.11 Small‐Angle X‐Ray Scattering

SAXS data were collected at the BL19U2 beamline of the National Facility for Protein Science Shanghai (NFPSS) at the Shanghai Synchrotron Radiation Facility (SSRF). The incident X‐ray beam was set to 12 keV, corresponding to a wavelength (*λ*) of 0.103 nm. Scattered intensities were recorded using a PILATUS3×2 M detector (DECTRIS Ltd., Switzerland). A sample‐to‐detector distance of 2675 mm was used, providing access to a scattering vector *q* range of 0.08 to 4.5/nm. To minimize background scattering, samples purified by size‐exclusion chromatography were continuously flowed through a quartz capillary with an inner diameter of 1.5 mm. Each scattering pattern was acquired with an exposure time of 1 s, and 20 consecutive frames were collected and averaged to improve signal‐to‐noise ratio as well as to assess radiation‐induced sample damage. Two‐dimensional (2D) scattering images were converted to one‐dimensional (1D) SAXS curves by azimuthal averaging after solid angle correction, normalized to the intensity of the transmitted X‐ray beam, and background‐subtracted using the software BioXTAS RAW (version 2.3.0) [61].

### 4.12 Modeling of SAXS Data

SAXS data were analyzed using both shape‐independent and shape‐dependent approaches. For model‐free analysis, the radius of gyration (*R_g_*) was determined via Guinier fitting (Equation 1) in the low‐*q* region (*qR_g_* < 1.3) using the PRIMUS module of the ATSAS software package.

(1) $$I\left(q\right)=\mathrm{scale}\cdot \exp\left[\frac{-Q^{2}R_{g}^{2}}{3}\right]+\mathrm{background}$$

To interpretunicode the scattering data in terms of a specific shape, a cylindrical form factor was fitted to the profiles using SasView software (version 5.0.6). The data were analyzed with the Cylinder model, employing a least‐squares minimization routine to extract structural parameters such as the cylinder radius and length (Equation 2).

(2) $$I\left(q,\alpha \right)=\frac{scale}{V}\;F^{2}\left(q,\alpha \right)+\mathrm{background}$$

where $F\left(q,\alpha \right)=2\left(\Delta \rho \right)V\frac{\sin(\frac{1}{2}qL\cos\alpha )}{\frac{1}{2}qL\cos\alpha }\frac{J_{1}\left(qR\sin\alpha \right)}{qR\sin\alpha }$

Here, α represents the angle between the cylinder axis and the scattering vector *q*; *V* = πR^2^L is the cylinder volume, with *L* and *R* denoting its length and radius, respectively; and Δ*ρ* (contrast) corresponds to the difference in scattering length density between the scatterer and the solvent. *J*_1_ refers to the first‐order Bessel function. The Sbp9^Δ^ showed polydispersity in both oxidizing and reducing conditions. To account for the size polydispersity, a Gaussian distribution function (Equation 3) was incorporated into the model.

(3) $$f\left(x\right)=\frac{1}{\mathrm{Norm}}\exp\left[-\frac{{\left(x-\bar{x}\right)}^{2}}{2\sigma^{2}}\right]$$

Here, $\bar{x}$ is the mean of the distribution, and Norm is the normalization factor determined during numerical fitting. The polydispersity index (PD) is defined as *σ*/$\bar{x}$.

### 4.13 Sbp9^Δ^ Self‐Assembly Under Physiologically Relevant Conditions

To mimic physiological oxidative stress, a glucose‐glucose oxidase (Glu‐GOx) cascade was utilized. Specifically, Sbp9^Δ^ protein (0.6 mg/mL) was incubated with glucose oxidase (Gox, 0.1 U/mL) and glucose (5 mM) for 10 min. To simulate a reducing environment, reduced glutathione (GSH) was employed at a concentration of 2 mM, and Sbp9^Δ^ (0.6 mg/mL) was incubated with GSH for 30 min. Time‐dependent changes in solution turbidity and coating morphology were subsequently evaluated.

### 4.14 Water Contact Angle (WCA)

Coatings were prepared on mica, glass, polystyrene (PS), polyethylene (PE), titanium (Ti), and aluminum (Al) substrates. The static WCA of each coating was measured using a DSA instrument (Krüss, Germany). A 10 µL droplet of ultrapure water was deposited onto the coating surface, and the contact angle was recorded immediately. For each coating, measurements were taken at three randomly selected locations, and the average value was reported. Uncoated substrates were tested under identical conditions as controls.

### 4.15 Nano‐Scratch Measurement

The adhesion strength of Sbp9^Δ^ coatings to various substrates was determined using UNHT^3^ (Anton Paar, Austria) equipped with a diamond flat‐punch tip (radius: 100 µm). Scratch tests were performed under a linearly increasing load from 0 to 2 N over a scratch length of 500 µm, at a loading rate of 2 N/min. The critical load, corresponding to coating delamination, was determined from the scratch profile. Three independent scratches were performed on each coated substrate, and the average critical load was calculated.

### 4.16 X‐Ray Photoelectron Spectroscopy (XPS)

The surface elemental composition of coatings deposited on glass, PS, and Ti was characterized by XPS. Measurements were performed using a PHI5000 VersaProbe III spectrometer (Ulvac‐Phi, Japan) equipped with a monochromatic Al Kα X‐ray source (1486.6 eV). An analysis area of 200 µm was defined by the X‐ray beam size. Survey spectra were acquired at an electron takeoff angle of 45°, with a pass energy of 224 eV and an energy step size of 1.0 eV. To obtain detailed chemical state information, high‐resolution spectra of the C 1s region were recorded using a pass energy of 69 eV and a step size of 0.1 eV. All spectral data were processed using Origin software (Version 9.1).

### 4.17 1,1‐Diphenyl‐2‐Picrylhydrazyl (DPPH) Radical Scavenging

Antioxidant activity of Sbp9^∆^ was quantified using the DPPH radical scavenging method. 100 µL of each sample at various concentrations (0.1%‐3%) was mixed with 100 µL of 0.2 mM DPPH solution (dissolved in methanol) in a 96‐well microplate. The mixture was incubated in the dark at room temperature for 30 min. Absorbance was then measured at 517 nm using a microplate reader (*n* = 3).

### 4.18 Characterization of Adsorption Performance of Coatings

The wet‐state adsorption performance of coatings deposited on glass, PS, and Al substrates was evaluated quantitatively under the following conditions: (i) exposure to flowing tap water for 30 min; (ii) immersion in flowing water for 60 days, during which the coated samples were placed in a beaker with a magnetic stirrer operating at 300 rpm, and samples were retrieved for staining analysis on days 1, 30, and 60. Coating morphology was examined using optical microscopy, and the coated area was quantified using ImageJ software (Version 2.1.0).

The adhesion force of the protein coating was measured using an NTEGRA Prima atomic force microscope (NT‐MDT, Russia). A cantilever with a spring constant of 0.03 N/m was employed, with a 10 µm‐radius glass microsphere attached to its tip. Prior to measurement, the microsphere‐functionalized probe was immersed in 40 µL of protein solution for 20 min to allow protein adsorption onto the microsphere surface. During force measurement, the functionalized probe was approached toward a thoroughly cleaned glass surface at a constant speed of 0.6 µm/s until contact was made. The force‐distance curve was then recorded in real time during retraction.

### 4.19 In Vivo Skin Penetration

To evaluate the transdermal permeability of the Sbp9^Δ^ coating, FITC‐labeled Sbp9^Δ^ was applied at concentrations of 1% and 3% (w/v). The dialysate collected after FITC‐labeling purification was used as a negative control to exclude interference from solution fluorescence and skin autofluorescence. Following application to the dorsal skin, mice were sacrificed at 1, 12, and 24 h. The treated skin was harvested and processed for frozen sectioning. Fluorescence distribution across different skin layers was visualized using confocal laser scanning microscopy (Zeiss LSM 980, Germany).

### 4.20 Hemocompatibility of Sbp9^Δ^ Coating

Mouse blood was collected from the orbital venous plexus, and the red blood cells were isolated by centrifugation and washed three times with normal saline (NS). The washed cells were resuspended in NS to obtain a 5% (v/v) erythrocyte suspension. Subsequently, 500 µL of Sbp9^Δ^ solution, sterile water (positive control), or NS (negative control) was mixed with 500 µL of the erythrocyte suspension, and the mixtures were incubated at 37°C for 1 h. After incubation, the samples were centrifuged, and the absorbance of the supernatants was measured at 540 nm (*n* = 3).

### 4.21 Dermal Safety of Sbp9^Δ^ Coating

The in vivo dermal safety of Sbp9^Δ^ coating was evaluated through skin irritation tests conducted in accordance with OECD Guideline 404. Normal saline and the Sbp9^Δ^ coating were separately applied topically to the dorsal skin of SD rats once daily for 7 consecutive days. Skin erythema or edema was evaluated daily and scored according to standard criteria. After 7 days, the skin samples were collected for H&E staining to evaluate inflammatory cell infiltration.

### 4.22 In Vivo Evaluation of Coatings for Androgenetic Alopecia (AGA) Treatment

All experimental procedures were approved by the Animal Ethics Committee of Ocean University of China (Approval No. OUC‐AE‐2025‐284). The hair regeneration efficacy of Sbp9^Δ^ coatings was evaluated using male C57BL/6 mice (7 weeks old, Huachuang Sino Co., Ltd., Taizhou, China). A dorsal area of approximately 9 cm^2^ (3 cm × 3 cm) in the telogen phase was gently shaved with an electric clipper and subsequently depilated using hair removal cream. The mice were randomly divided into four groups: Healthy group (saline), AGA group, MXD group (5% minoxidil), and coating group (3% Sbp9^Δ^ mixed with 0.1 mM Ca^2+^). The AGA model was established by subcutaneous injection of testosterone propionate (TP) at a dose of 10 mg/kg. TP injection was maintained throughout the experimental period to sustain stable androgen levels. Concurrently, MDX (0.1 mL/cm^2^) or Sbp9^Δ^ coating (0.1 mL/cm^2^) was applied daily. Digital photographs of skin and hair were taken periodically. On day 20 post‐depilation, the diameter of regenerated hair shafts was measured under a Motic AE2000 microscope.

### 4.23 Skin Tissue Histology

On day 15, animals were sacrificed for tissue collection. Skin samples from the depilated area were processed for hematoxylin and eosin (H&E) staining and for the detection of Ki67, CD31, and dihydroethidium (DHE) fluorescence. For paraffin sections, tissues were fixed in 4% (w/v) formaldehyde, dehydrated, embedded, and sectioned at 8 µm thickness for H&E and immunohistochemical staining of Ki67 and CD31. For frozen sections, tissues were embedded in OCT compound, frozen, and cryo‐sectioned at 10 µm thickness. The sections were incubated with DHE (Sparkjade) followed by DAPI (Beyotime) and imaged using a laser scanning confocal microscope (Zeiss LSM980, Germany). Additionally, skin homogenates were prepared from the depilated area on day 15 by cutting the tissue into pieces and homogenizing in PBS using steel beads. The homogenate was centrifuged at 12 000 × *g* for 30 min, and the supernatant was collected for biochemical assays. The levels of malondialdehyde (MDA), glutathione (GSH), and superoxide dismutase (SOD) were determined using corresponding assay kits (MDA assay kit, Total Glutathione Assay Kit, and Total SOD Assay Kit with WST‐8, all from Beyotime).

### 4.24 RNA Sequencing

To elucidate the mechanism by which the Sbp9^Δ^ coating promotes hair regrowth, the skin of the healthy group, AGA group, and coating group was collected on day 15. Total RNA was extracted, and the sequencing platform of illumina Novaseq^TM^ X plus (Novogene Co., Ltd., China) was applied to obtain the gene expression profiles. PCA and Pearson correlation analysis were performed using the OmicShare tools. The DEGs in the comparison of coating versus AGA were subjected to GO enrichment analysis and KEGG pathway enrichment analysis.

### 4.25 RT‐qPCR Analysis

cDNA was synthesized from total RNA per sample using *Evo M*‐*MLV* RT Mix Kit with gDNA Clean for qPCR (AG11728, ACCURATE BIOTECHNOLOGY (HUNAN) Co., Ltd., ChangSha, China). Quantitative real‐time PCR (RT‐qPCR) was performed using a LightCycler 480 II real‐time PCR system (Roche, Switzerland) and SYBR Green Premix Pro Taq HS qPCR Kit (AG11701, ACCURATE BIOTECHNOLOGY(HUNAN) Co., Ltd., ChangSha, China). GAPDH was used as the internal reference gene. Each assay was performed in triplicate. The primer sequences used for RT‐qPCR analysis were listed in Table S3.

### 4.26 Statistics and Data Analysis

All data were presented as the mean ± standard deviation. For comparisons between two groups, a two‐tailed Student's *t*‐test was used, while one‐way ANOVA with Tukey's multiple comparison test was applied for multiple group comparisons (SPSS 22.0).

## Author Contributions

L.W. performed conceptualization, funding acquisition, and writing – original draft. J. Y. performed sample collection, investigation, and formal analysis. X.J. performed formal analysis, visualization, and writing – original draft. Z. Z. performed formal analysis and data curation. H.W. performed validation and methodology. N.L. performed supervision and conceptualization. X.C. performed project administration, supervision, and funding acquisition. W.L. performed conceptualization, project administration, resources, and funding acquisition.

## Conflicts of Interest

The authors declare no conflicts of interest.

## Supporting information




**Supporting File 1**: advs76497‐sup‐0001‐SuppMat.docx.

**Supporting File 2**: advs76497‐sup‐0002‐MovieS1.mp4.

## Data Availability

The data that support the findings of this study are available from the corresponding author upon reasonable request.
