# Supplementary material for: A Disulfide‐Sticker Strategy for Marine Adhesive Coatings: From Deciphering Self‐Assembly Mechanism to Functional Application in Hair Regeneration
Source: Adv Sci (Weinh). 2026 Jul 9;13(55):e76497. doi: 10.1002/advs.76497 (PMC13348336; doi:10.1002/advs.76497)
Supplement: Supplementary file 1 [file ADVS-13-e76497-s001.docx]

Supporting Information

**A Disulfide-Sticker Strategy for Marine Adhesive Coatings: From Deciphering Self-Assembly Mechanism to Functional Application in Hair Regeneration**

*Lulu Wang, Juan Yang, Xin Jiang, Zhanghui Zheng, Hongyu Wei, Na Li*, Xiangqiang Chu*, Weizhi Liu**

**E-mail:** [liuweizhi@ouc.edu.cn](mailto:liuweizhi@ouc.edu.cn); [xiangchu@cityu.edu.hk](mailto:xiangchu@cityu.edu.hk); lina02@sari.ac.cn

L. Wang, J. Yang, and X. Jiang contributed equally to this work.

**Figure S1**. Effect of EGFL repeats on R_h_ during Ca^2+^-induced self-assembly of recombinant proteins.

**Figure S2**. Effect of Ca^2+^ presence or absence on phase separation of Sbp9^∆^ protein. The dense phase is indicated by the white arrow.

**Figure S3**. Analysis of amino acid composition in Sbp9^∆^ protein. Cysteine is the most abundant residue, accounting for 17.6%.

**Figure S4**. Force-Distance curves of (a) Sbp9^∆^ coating, (b) H_2_O_2_-Sbp9^∆^ coating, and (c) DTT-Sbp9^∆^ coating.

**Figure S5.** Self-assembly and coating formation of Sbp9^Δ^ under physiological relevant redox conditions. (a) Time-dependent turbidity changes induced by 1 mM Ca^2+^ over a 30-min period. (b) Representative morphologies of coatings formed in the presence of 5 mM Ca^2+^. Scale bar = 200 µm.

**Figure S6**. DSC thermograms of Sbp9^∆^ under different redox conditions.

**Figure S7**. Water contact angle (WCA) and atomic force microscope (AFM) images of bare substrates prior to Sbp9^∆^ coating treatment. PS, polystyrene. PE, polyethylene. Ti, titanium. Al, aluminum. Scale bar = 1 µm.

**Figure S8**. Quantification of the coating area on various substrates after immersion in flowing water environments for 1, 30, and 60 days.

**Figure S9**. Force-Distance curves of (a) BSA coating, (b) Cell-Tak coating, (c) Sbp9^∆^ coating.

**Figure S10**. Nano-scratch evaluation of the adhesion performance of Sbp9^∆^ coatings on glass, polystyrene (PS), and titanium (Ti) substrates. Scale bar = 100 µm.

**Figure S11**. XPS analysis of Sbp9^∆^ coatings on various substrates of (a) Glass, (b) PS, and (c) Ti. Grey curves represent XPS results on bare substrates, and blue curves correspond to Sbp9^∆^ coated substrates.

**Figure S12**. Representative fluorescence microscopy images of longitudinal dorsal skin treated with 1% and 3% FITC-labeled Sbp9^∆^ coatings at 1 h, 12 h, and 24 h post-administration. Scale bar = 200 µm.

**Figure S13**. Quantitative analysis of the fluorescence intensity for 1% and 3% FITC-labeled Sbp9^∆^ coatings.

**Figure S14**. Quantification of skin thickness based on H&E staining of dorsal skin tissue in each group.

**Figure S15**. (a) Hemolysis analysis of Sbp9^∆^ coatings. (b) H&E staining of the dorsal skin of SD rats after topical application with Sbp9^∆^ coating. Scale bar = 400 μm.

**Figure S16**. The H&E staining of heart, liver, spleen, lung, and kidney tissues. Scale bar = 200 μm.

**Figure S17**. (a) Gene Ontology (GO) enrichment analysis, and (b) Kyoto Encyclopedia of Genes and Genomes (KEGG) pathway enrichment analysis showing the top-ranked down-regulated signaling pathways in the coating group compared to the AGA group.

**Figure S18**. RT-qPCR verification of (a) *Lef1*, *Bambi*, *Wnt5a*, *Tcf7*, *Krt71*, *Shh*, *Tyrp1*, *Dct*, and (b) *Mylk4*, *Trdn*.

**Table S1.** The *R_g_*, radius and length of Sbp9^Δ^ under different redox conditions.

|  | ***R_g_* (Å)** | **Radius (Å)** | **Length (Å)** | **Polydispersity** |
| --- | --- | --- | --- | --- |
| Sbp9^∆^ | 143.99 ± 3.45 | 45.37 ± 5.03 | 172.36 ± 10.23 | 0.085 |
| Oxidized-Sbp9^∆^ | 166.93 ± 11.9 | 40.62 ± 4.15 | 198.28 ± 17.28 | 0.172 |
| Reduced-Sbp9^∆^ | 133.29 ± 1.11 | 35.93 ± 5.48 | 95.31 ± 7.33 | 0.156 |

**Table S2.** Dermal reactions following application of Sbp9^Δ^ coatings (n = 5).

| **Group** | **Total irritation score** | | | | | | | **Irritation index** | **Interpretation** |
| --- | --- | --- | --- | --- | --- | --- | --- | --- | --- |
|  | Day1 | Day2 | Day3 | Day4 | Day5 | Day6 | Day7 |  |  |
| Control | 0 | 0 | 0 | 0 | 0 | 0 | 0 | 0 | No irritation |
| Coating | 0 | 0 | 0 | 0 | 0 | 0 | 0 | 0 | No irritation |

**Table S3.** Primer sequences used for qRT-PCR.

| **Primer** | **Sequence (5'-3')** |
| --- | --- |
| *GAPDH-F* | GTGGCAAAGTGGAGATTGTTG |
| *GAPDH-R* | CGTTGAATTTGCCGTGAGTG |
| *Lef1-F* | CAAGGGACCCTCCTACTCCA |
| *Lef1-R* | ATCCCGGAGAAAAGTGCTCG |
| *Bambi-F* | ATTGCTGGCGGACTGATCTT |
| *Bambi-R* | CAACTTTGCAACTTGCCCCT |
| *Wnt5a-F* | AAAGGGAACGAATCCACGCT |
| *Wnt5a-R* | CAGCACGTCTTGAGGCTACA |
| *Tcf7-F* | AGGTGGCATGCACTATCTCG |
| *Tcf7-R* | CCGCCTCTTCTTCTTTCCGT |
| *Krt71-F* | GATGCGGCTTATGCCAACAA |
| *Krt71-R* | TCTCGGCTTCGAACAGACAC |
| *Shh-F* | GCACCATTCTCATCAACCGGG |
| *Shh-R* | CCCTCGCTTCCGTTGCAGA |
| *Tyrp1-F* | GCACACTTTCACTGATGCGG |
| *Tyrp1-R* | GTGCGTTTTCCAACGGGAAG |
| *Dct-F* | CTGGCAATGAGTCCTTTGCG |
| *Dct-R* | TCCGACTAATCAGCGTTGGG |
| *Mylk4-F* | CTCCTGCCGCTCCATTTGAT |
| *Mylk4-R* | CCTCCTTGTCCTTTGCACCT |
| *Trdn-F* | TGCTGTGGAGGAGACAACAG |
| *Trdn-R* | GCATCCTCATCATCTTCGTCAC |
